# Region-specific alteration of histone modification by LSD1 inhibitor conjugated with pyrrole-imidazole polyamide

**DOI:** 10.18632/oncotarget.25451

**Published:** 2018-06-29

**Authors:** Kokiladevi Alagarswamy, Ken-Ichi Shinohara, Shihori Takayanagi, Masaki Fukuyo, Atsushi Okabe, Bahityar Rahmutulla, Natsumi Yoda, Rui Qin, Naoki Shiga, Masahiro Sugiura, Hiroaki Sato, Kazuko Kita, Takayoshi Suzuki, Tetsuhiro Nemoto, Atsushi Kaneda

**Affiliations:** ^1^ Department of Molecular Oncology, School of Medicine, Chiba University, Chiba, Japan; ^2^ Department of Pharmaceutical Chemistry, Graduate School of Pharmaceutical Sciences, Chiba University, Chiba, Japan; ^3^ Department of Chemistry, Graduate School of Medical Science, Kyoto Prefectural University of Medicine, Kyoto, Japan

**Keywords:** epigenome, histone modification, lysine-specific demethylase-1 inhibitor, pyrrole imidazole polyamide

## Abstract

Epigenome regulates gene expression to determine cell fate, and accumulation of epigenomic aberrations leads to diseases, including cancer. NCD38 inhibits lysine-specific demethylase-1 (LSD1), a histone demethylase targeting H3K4me1 and H3K4me2, but not H3K4me3. In this study, we conjugated NCD38 with a potent small molecule called pyrrole (Py) imidazole (Im) polyamide, to analyze whether targets of the inhibitor could be regulated in a sequence-specific manner. We synthesized two conjugates using β-Ala (β) as a linker, i.e., NCD38-β-β-Py-Py-Py-Py (NCD38-β_2_P_4_) recognizing WWWWWW sequence, and NCD38-β-β-Py-Im-Py-Py (NCD38-β_2_PIPP) recognizing WWCGWW sequence. When RKO cells were treated with NCD38, H3K4me2 levels increased in 103 regions with significant activation of nearby genes (*P* = 0.03), whereas H3K4me3 levels were not obviously increased. H3K27ac levels were also increased in 458 regions with significant activation of nearby genes (*P* = 3 × 10^−10^), and these activated regions frequently included GC-rich sequences, but less frequently included AT-rich sequences (*P* < 1 × 10^−15^) or WWCGWW sequences (*P* = 2 × 10^−13^). When treated with NCD38-β_2_P_4_, 234 regions showed increased H3K27ac levels with significant activation of nearby genes (*P* = 2 × 10^−11^), including significantly fewer GC-rich sequences (*P* < 1 × 10^−15^) and significantly more AT-rich sequences (*P* < 1 × 10^−15^) compared with NCD38 treatment. When treated with NCD38-β_2_PIPP, 82 regions showed increased H3K27ac levels, including significantly fewer GC-rich sequences (*P* = 1 × 10^−11^) and fewer AT-rich sequences (*P* = 0.005), but significantly more WWCGWW sequences (*P* = 0.0001) compared with NCD38 treatment. These indicated that target regions of epigenomic inhibitors could be modified in a sequence-specific manner and that conjugation of Py-Im polyamides may be useful for this purpose.

## INTRODUCTION

Epigenetic modifications, e.g., DNA methylation and histone modification, have been identified as key epigenetic alterations that regulate gene expression; hence, their dysregulation is often associated with substantial diseases [1]. Cancer is known to arise through accumulation of epigenetic and genetic aberrations, and many tumor-suppressor genes are epigenetically inactivated in cancer, leading to tumor progression, invasion, and metastasis [2–4].

For modifications at histone tails, methylation at the lysine 4 residue of histone H3 (H3K4me) reflects transcriptional competency, whereas those at lysines 9 and 27 (H3K9me and H3K27me, respectively) are components of the repressive chromatin structure [5]. High levels of H3K4 trimethylation (H3K4me3) or H3K27 acetylation (H3K27ac) at the promoters are implicated in transcriptional activation, whereas H3K27 trimethylation (H3K27me3) is correlated with gene repression and silencing [6]. The establishment of appropriate histone modification patterns is essential for normal development and tissue differentiation, whereas dysregulation of these modulators and aberrations of histone modifications are associated with cancer development [7, 8].

Lysine-specific demethylase-1 (LSD1) was the first histone demethylase enzyme to be identified for the selective demethylation of H3K4 monomethylation (H3K4me1) and dimethylation (H3K4me2), but not H3K4me3 [9]. LSD1 suppresses the expression of genes through flavin adenine dinucleotide (FAD)-dependent enzymatic oxidation processes [10]. In addition, LSD1 has been reported to be aberrantly upregulated in many human cancer types, such as prostate, breast, and lung cancer as well as neuroblastoma and leukemia [11–15]. Suzuki and colleagues recently developed the potent LSD1 inhibitor NCD38 [16, 17], which consists of a lysine moiety for enzyme selectivity and a trans-phenylcyclopropylamine for FAD alkylation [18, 19]. NCD38 reportedly inhibits the cellular growth of human leukemia, lung cancer and glioma stem cells and alters the genome-wide chromatin status [17, 20, 21].

Although over 170 anticancer drugs have been approved by the US Food and Drug Administration (FDA), only six agents targeting epigenetic processes have been approved, including two DNA methyltransferase (DNMT) inhibitors (azacytidine and decitabine) and four histone deacetylase (HDAC) inhibitors (vorinostat, romidepsin, belinostat, and panobinostat) [22, 23]. These agents, however, reprogram the epigenome broadly and randomly; thus, unfavorable side effects can occur, along with the antitumor effects [24, 25]. A number of epigenetic agents are under development or in clinical trials, including inhibitors against histone methylases and demethylases in addition to DNMT and HDAC inhibitors to obtain better efficacy and low toxicity.

Another approach is the development of precisely tunable small molecules possessing both the ability for recognition of selective genomic regions and for altering epigenetic modifications. Pyrrole (Py) imidazole (Im) polyamide is a cell-permeable small molecule which binds the minor groove of double strand DNA (dsDNA) in 2:1 ligand to DNA stoichiometries with the following rules. Since Py and Im recognizes A/C/T and G, respectively, Py/Py pair and Im/Py pair selectively binds to A/T pair and G/C pair in dsDNA, respectively [26–28]. β-Ala (β) behaves like Py; Py/β pair also selectively binds to A/T pair in dsDNA. Py-Im polyamide reportedly acts as an artificial gene silencer with site-selective regulation by interfering with the interactions between transcription factors and their DNA binding sites [29–32]. Moreover, conjugation of Py-Im polyamide with a functional molecule can confer the functional molecule with sequence selectivity and nuclear accumulation, as is observed for DNA alkylating agents and fluorophores, resulting in unique bioactivities and tools [28, 33, 34].

In this study, we applied the technologies of Py-Im polyamides to activate genomic regions in a sequence-specific manner by conjugation with an epigenetic inhibitor. We conjugated NCD38 with two different polyamides, and analyzed region-selective activation by genome-wide methods.

## RESULTS

### ChIP-seq and RNA-seq analyses of NCD38-treated cells

**Figure 1:**
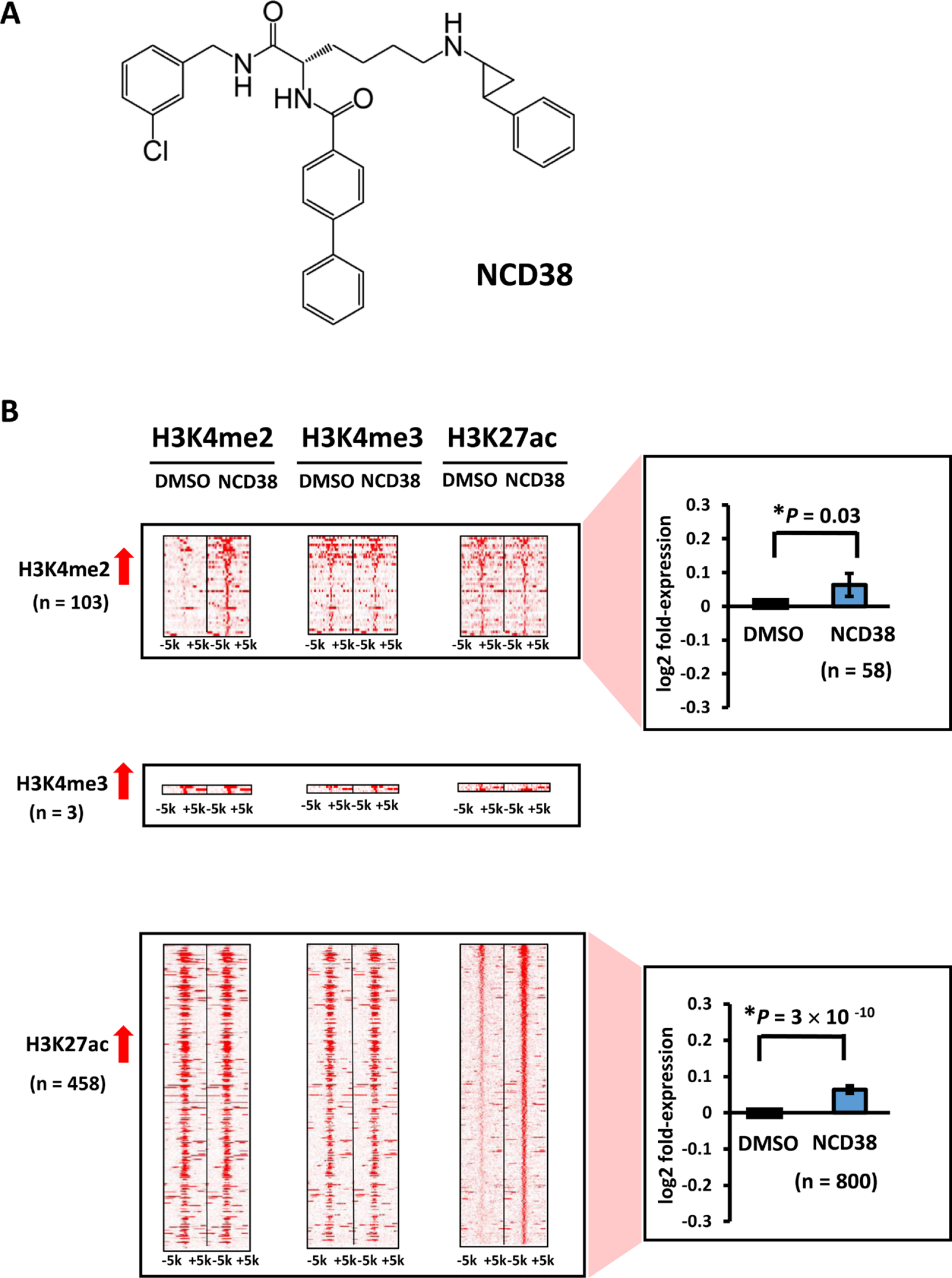
Alteration of histone modification by NCD38 treatment (**A**) Chemical structure of the LSD1 inhibitor NCD38. (**B**) Heat maps showing the read densities of ChIP-seq within ± 5 kb around the center position of ChIP-seq peaks. Whereas increase of H3K4me3 level was hardly observed, 103 and 458 regions showed >3-fold increase of H3K4me2 and H3K27ac levels, respectively. Expression of genes nearest to the H3K4me2 and H3K27ac peaks were significantly upregulated (*P* = 0.03 and *P* = 3 × 10^−10^, respectively, *t*-test).

Initially, we carried out genome-wide analysis for histone modification alterations induced by the LSD1 inhibitor NCD38 (Figure 1A). We performed ChIP-seq analysis for H3K4me2, H3K4me3, and H3K27ac, and RNA-seq analysis for RKO cells treated with NCD38 for 30 days. Although H3K4me3 levels were barely increased (three regions only), 103 and 458 regions showed more than 3-fold increases in H3K4me2 and H3K27ac levels, respectively, in NCD38-treated cells compared with DMSO-treated cells (Figure 1B). RNA-seq analysis revealed that the expression levels of genes nearest to the ChIP-seq peaks of H3K4me2 and H3K27ac were significantly increased in NCD38-treated cells compared with DMSO-treated cells (*P* = 0.03 and *P* = 3 × 10^–10^, respectively). These findings indicated that NCD38 treatment could increase H3K4me2 and H3K27ac levels, resulting in upregulation of genes around the activated regions.

### GC-rich regions preferentially activated by NCD38

**Figure 2:**
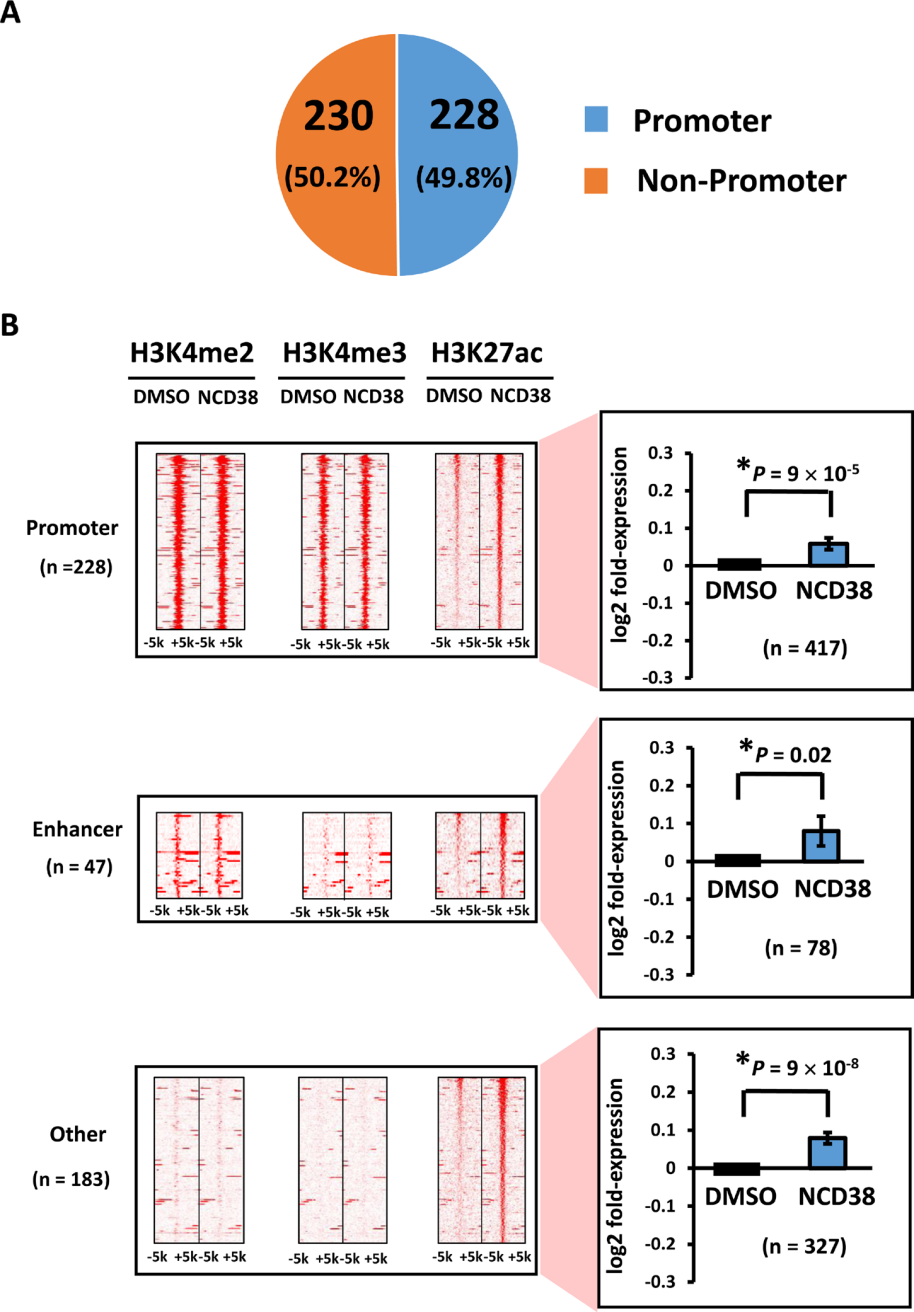
Distribution of H3K27ac-increased regions following NCD38 treatment (**A**) Pie chart for the distribution of H3K27ac peaks. About half of H3K27ac-increased regions are distributed in promoter regions. (**B**) Heat maps showing read densities of H3K4me2, H3K4me3, and H3K27ac within ± 5 kb around H3K27ac-increased regions in promoter, enhancer, and other regions. Genes nearest to the H3K27ac peaks in promoter, enhancer, and other regions were significantly upregulated (*P* = 9 × 10^−5^, *P* = 0.02, and *P* = 9 × 10^−8^, respectively).

Next, we analyzed features of activated regions using 458 H3K27ac-increased regions, since H3K27ac is a common active mark for promoter (i.e., H3K4me3-positive) and enhancer (i.e., H3K4me2-positive but H3K4me3-negative) regions. About half of the regions (228/458, 49.8%) were located on promoter regions, whereas 47 and 183 regions were distributed in enhancers and other regions, respectively (Figure 2A). Genes nearest to these H3K27ac peaks included 417 (promoter), 78 (enhancer), and 327 genes (other), and their expression levels were significantly increased (*P* = 9 × 10^−5^, *P* = 0.02, and *P* = 9 × 10^−8^, respectively), compared with those of DMSO-treated cells (Figure 2B).

**Figure 3:**
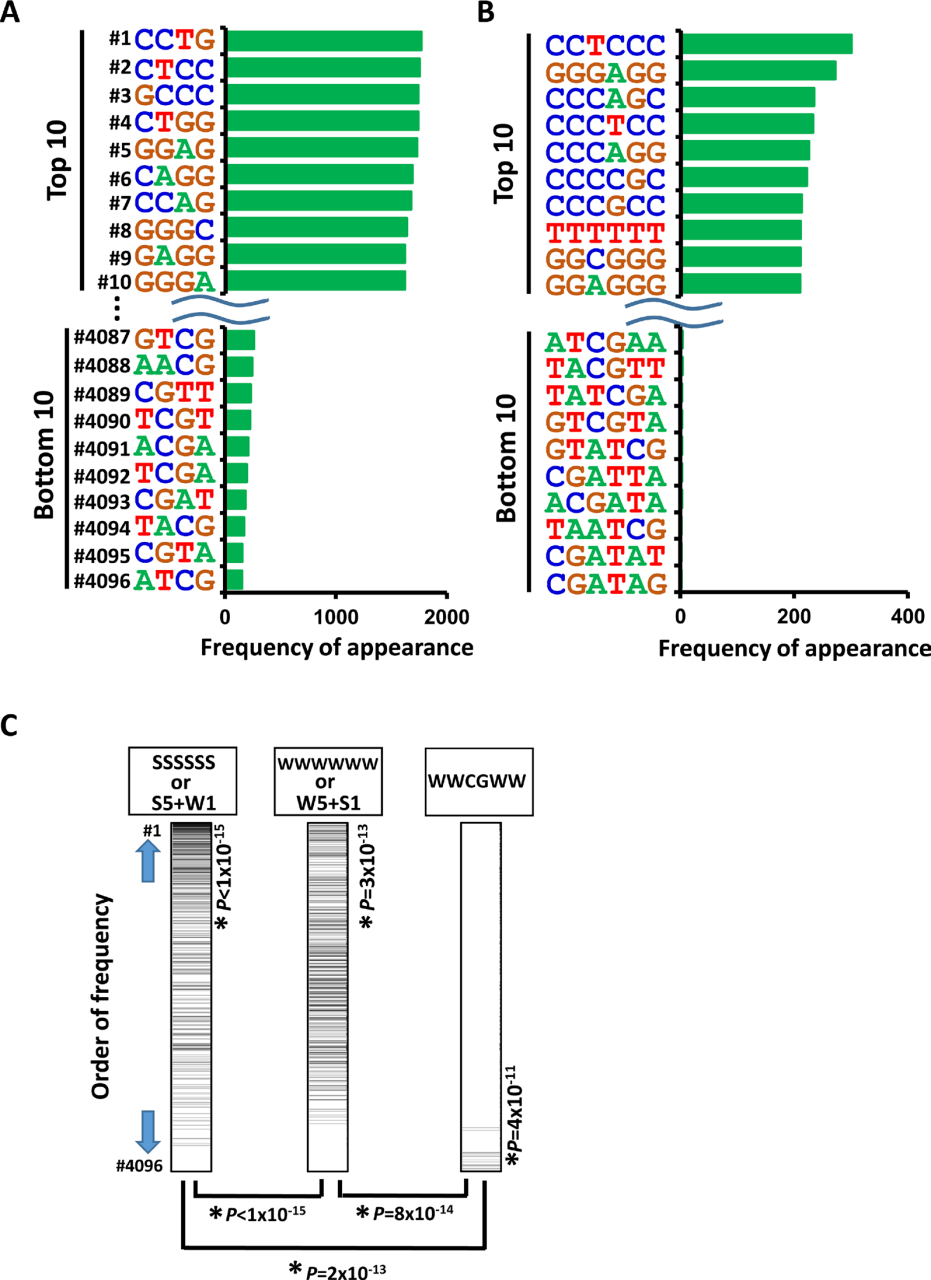
Appearance of DNA sequences in regions activated by NCD38 Frequencies of appearance of 4-bp (**A**) and 6-bp (**B**) DNA sequences within 250 bp from the center of the increased H3K27ac peaks are shown. GC-content of top 10 4-bp sequences was as high as 80% ± 3% (A), and that of top 10 6-bp sequences was also as high as 80% ± 9% (B). (**C**) Less frequent appearance of AT-rich and WWCGWW sequences. Total of 4,096 6-bp sequences were sorted by the order of frequency of appearance, with the most frequent sequence at the top (*#1*) and the most infrequent sequence at the bottom (*#4096*). SSSSSS or 6-bp sequences including five S and one W (*left*) were significantly enriched to the upward (*P* < 1 × 10^−15^, Kolmogorov-Smirnov test), showing that regions with GC-rich sequences are likely activated. WWWWWW or 6-bp sequences including five W and one S (*middle*) were also significantly enriched to the upward (*P* = 3 × 10^−13^), but relatively downward compared with SSSSSS or 6-bp sequences including five S and one W (*P* < 1 × 10^−15^), showing that AT-rich regions are less likely activated than GC-rich regions. WWCGWW (*right*) were significantly enriched to the downward (*P* = 4 × 10^−11^), and significantly to the downward compared with SSSSSS or 6-bp sequences including five S and one W (*P* = 2 × 10^−13^), showing that regions with WWCGWW sequences are unlikely activated.

**Figure 4:**
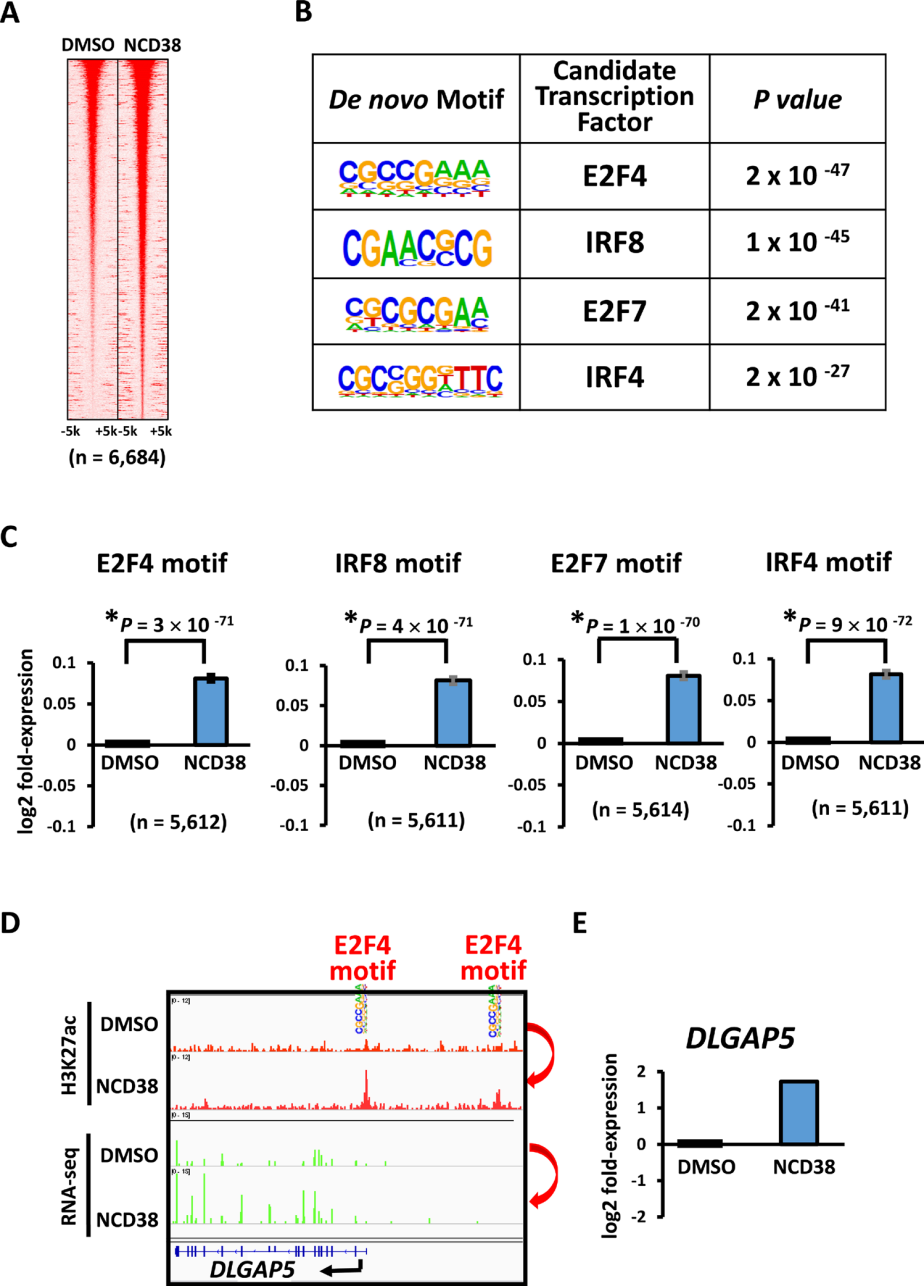
Enrichment of GC-rich motifs following NCD38 treatment (**A**) Heat maps showing read densities of H3K27ac within ± 5 kb around H3K27ac-increased regions. (**B**) *De novo* motif analysis. The motifs significantly enriched around H3K27ac-increased regions, E2F4, IRF8, E2F7, and IRF4, were all GC-rich sequences. (**C**) Expression of genes with the enriched motifs. Expression levels of genes nearest to the motives of E2F4, IRF8, E2F7, and IRF4, with increased H3K27ac levels, were significantly upregulated by NCD38. (**D**) Representative image of *DLGAP5*. H3K27ac level was increased at E2F4 motif site. (**E**) Representative RNA-seq results of *DLGAP5*. Expression of *DLGAP5* was increased.

Next, we analyzed the appearance of 4- and 6-bp DNA sequences within 250 bp from the center of the increased H3K27ac peaks. In good agreement with the preferential activation of promoter regions by NCD38 treatment, the top 10 sequences showed high GC contents, 80% ± 3% for 4-bp sequences and 80% ± 9% for 6-bp sequences (average ± standard error) (Figure 3A and 3B). In contrast, AT-rich sequences (*P* < 1 × 10^−15^), or sequences including WCGW such as WWCGWW (*P* = 2 × 10^−13^), appeared significantly less frequently (where W means A or T) (Figure 3C). We then performed *de novo* motif analysis to analyze the motifs significantly enriched around the H3K27ac-increased peaks. The top four significant motifs were E2F4, IRF8, E2F7, and IRF4 (*P* = 2 × 10^−47^, *P* = 1 × 10^−45^, *P* = 2 × 10^−41^, and *P* = 2 × 10^−27^, respectively), which were all GC-rich sequences (Figure 4A and 4B). Consistent with these findings, RNA-seq analysis revealed that the expression levels of genes nearest to these motifs with increased H3K27ac levels were significantly upregulated by NCD38 (*P* = 3 × 10^−71^, *P* = 4 × 10^−71^, *P* = 1 × 10^−70^, and *P* = 9 × 10^−72^, respectively; Figure 4C), e.g. *DLGAP5* activated by increase of H3K27ac at enhancers with E2F4 motif (Figure 4D and 4E).

### ChIP-seq and RNA-seq analyses of NCD38-β_2_P_4_-treated cells

**Figure 5:**
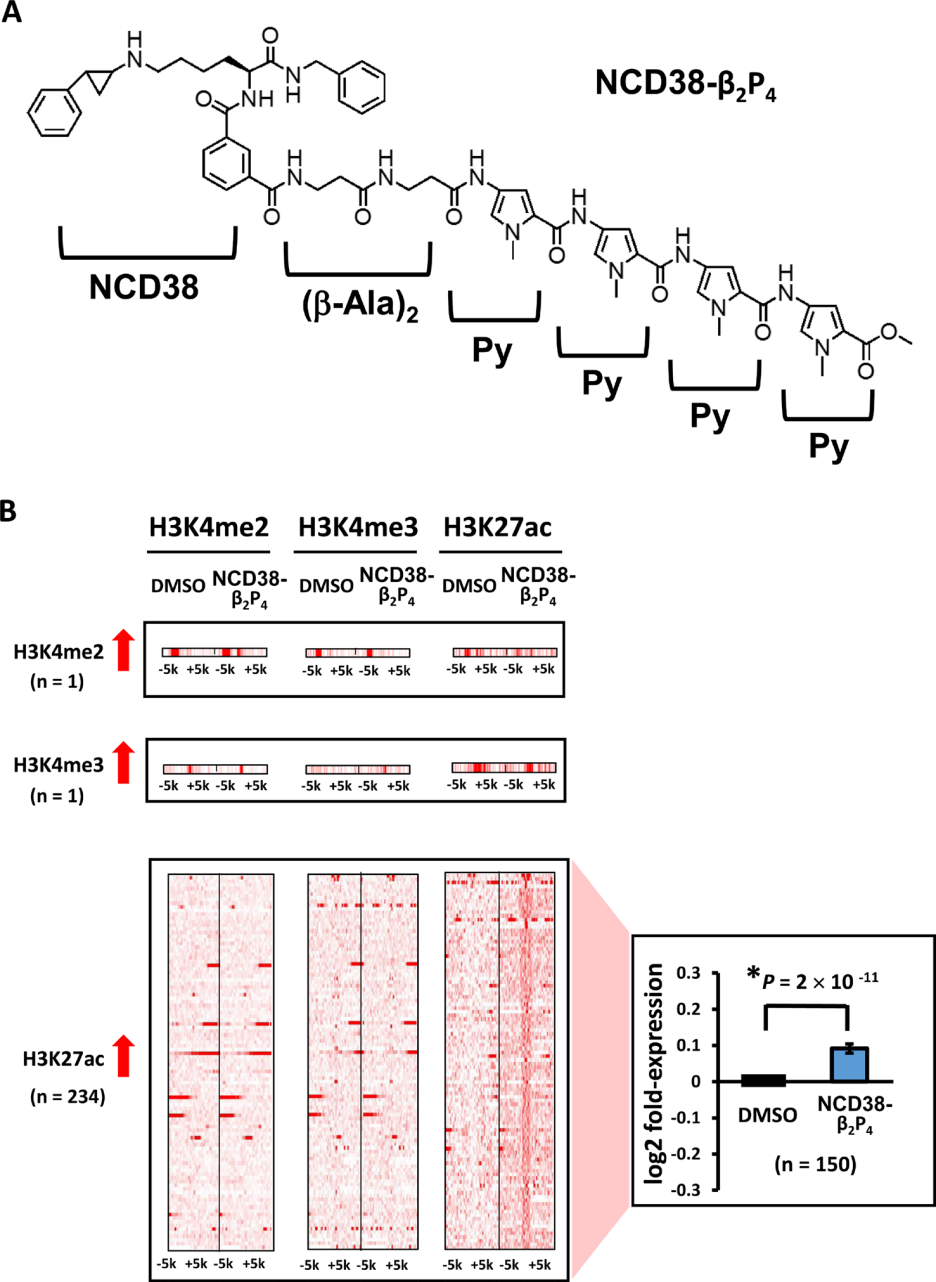
Alterations in histone modification by NCD38-β_2_P_4_ treatment (**A**) Chemical structure of NCD38-β_2_P_4_. A Py-Im polyamide Py-Py-Py-Py was conjugated with NCD38, with the dipeptide of β-alanine inserted. The conjugate recognizes WWWWWW sequences, and the specific binding to the sequence was confirmed (See Supplementary Figure 2). (**B**) Heat maps showing the read densities of ChIP-seq within ± 5 kb around the center position of ChIP-seq peaks. Less number of regions showed more than 3-fold increases in H3K27ac levels, compared with treatment by parental NCD38 (*See* Figure 1B). Expression of genes nearest to the H3K27ac peaks were significantly upregulated (*P* = 2 × 10 ^-11^, *t*-test).

Since regions containing AT-rich or WWCGWW sequences were less frequently activated by NCD38 treatment, we developed hybrid molecules containing NCD38 and Py-Im polyamides recognizing AT-rich and WWCGWW sequences, to analyze whether these less frequently activated regions could be targeted. First, a Py-Im polyamide Py-Py-Py-Py (P_4_) was conjugated with NCD38 with the dipeptide of β-alanine inserted as a linker, to develop NCD38-β-β-Py-Py-Py-Py (NCD38-β_2_P_4_) (Figure 5A). The cell permeability and nuclear localization of the Py-Im polyamide were confirmed using P_4_ conjugated with FITC (Supplementary Figure 1). Specific recognition of WWWWWW sequence by NCD38-β_2_P_4_ was confirmed; NCD38-β_2_P_4_ selectively bound to oligo DNA with WWWWWW sequence, but not to that with GWWWWG or WWCGWW (Supplementary Figure 2). The inhibitory activity of NCD38-β_2_P_4_ against histone demethylase LSD1 was confirmed to be similar to the parental NCD38 (Supplementary Figure 3A). It was also confirmed that both NCD38 and NCD38-β_2_P_4_ lacked inhibitory activity against HDAC (Supplementary Figure 3B).

**Figure 6:**
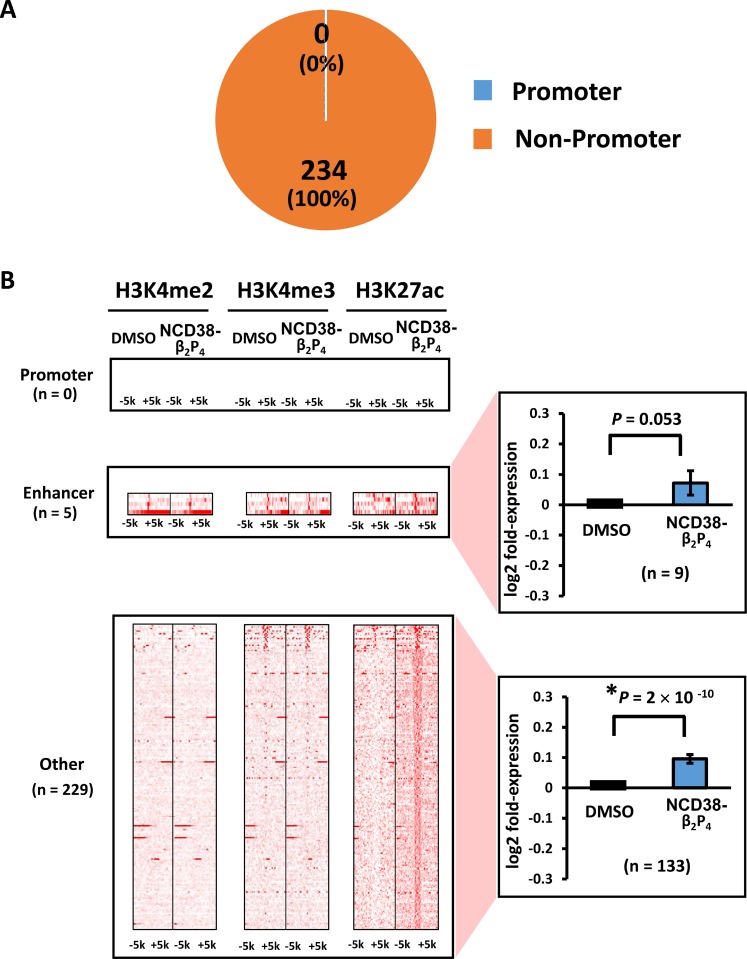
Distribution of H3K27ac-increased regions following NCD38-β_2_P_4_ treatment (**A**) Pie chart showing the distribution of H3K27ac peaks. All of the 234 regions were distributed in non-promoter regions. (**B**) Heat maps showing read densities of H3K4me2, H3K4me3, and H3K27ac within ± 5 kb around H3K27ac-increased regions in enhancers and other regions. Genes nearest to the H3K27ac peaks in other regions were significantly upregulated (*P* = 9 × 10^−10^), while those in enhancer regions tended to be upregulated (*P* = 0.053).

When RKO cells were treated with NCD38-β_2_P_4_ for 30 days, fewer regions (234) showed more than 3-fold increases in H3K27ac levels compared with 458 regions by NCD38 treatment (Figure 5B). RNA-seq analysis revealed that the expression levels of genes nearest to the H3K27ac-increased peaks were significantly increased in NCD38-β_2_P_4_-treated cells compared with DMSO-treated cells (*P* = 2 × 10^−11^). All of the 234 regions were distributed in non-promoter regions, including five enhancers and 229 other regions (Figure 6A and 6B), and there was no overlap of activated regions between NCD38 and NCD38-β_2_P_4_ treatment. Genes nearest to these H3K27ac peaks were upregulated (*P* = 0.053 and *P* = 9 × 10^−10^, respectively) compared with DMSO-treated cells.

For fear that these activation might be perhaps due to the effect of Py-Im polyamide itself, we treated RKO cells with P_4_ without conjugation of NCD38 for 30 days, and analyzed histone modification by ChIP-seq. Among the 234 regions with >3-fold increase of H3K27ac levels after 30-day treatment with the conjugate, none of them showed >3-fold or >1.5-fold increase after treatment with P_4_ (Supplementary Figures 4 and 5).

### AT-rich regions preferentially activated by NCD38-β_2_P_4_

**Figure 7:**
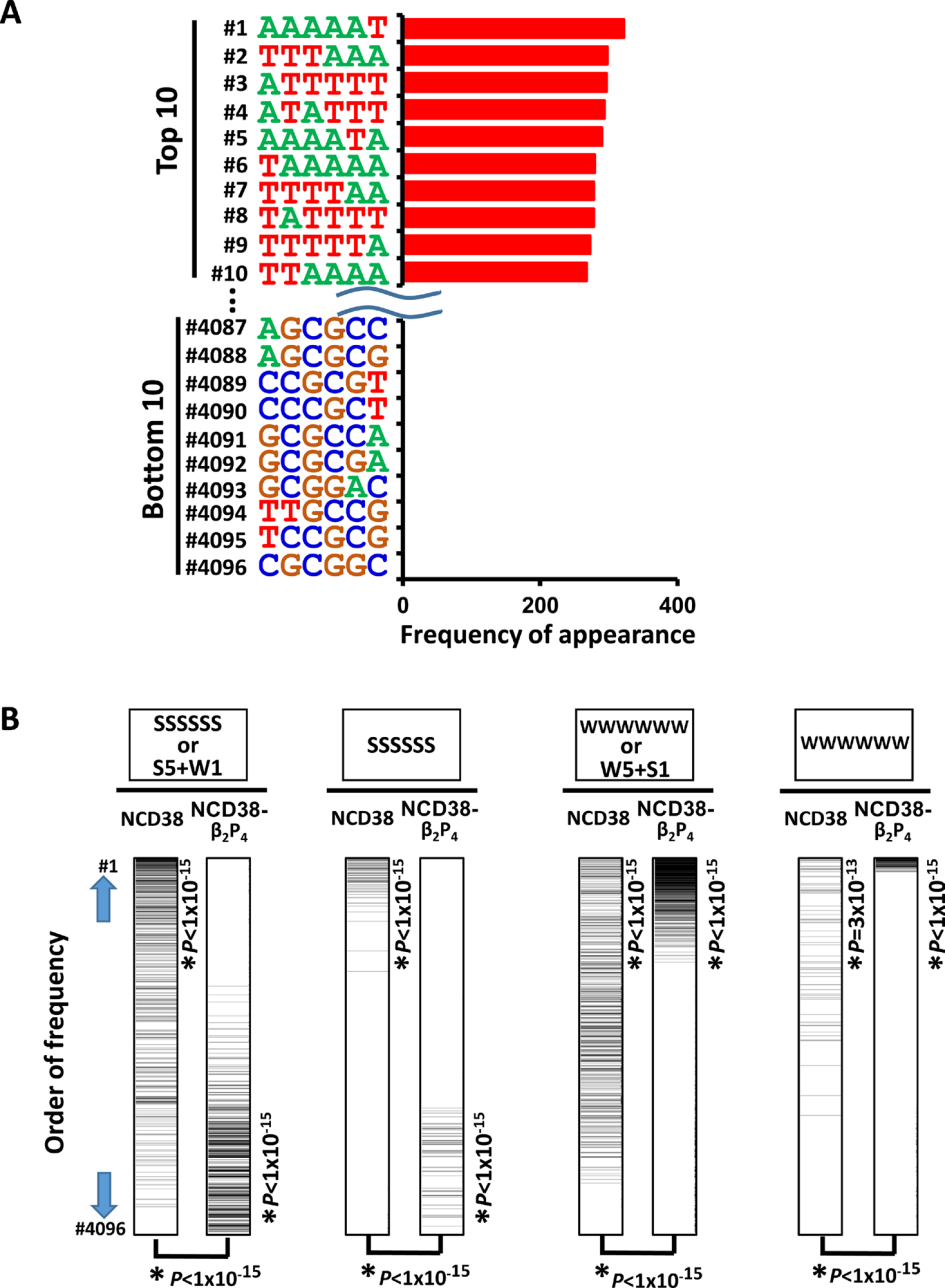
Appearance of DNA sequences in regions activated by NCD38-β_2_P_4_ (**A**) Frequencies of appearance of 6-bp sequences within 250 bp from the center of the increased H3K27ac peaks. All the top 10 sequences were WWWWWW. (**B**) Significant recognition of AT-rich sequences by NCD38-β_2_P_4_. Total of 4,096 6-bp sequences were sorted by the order of frequency of appearance. SSSSSS or 6-bp sequences including five S and one W (*the most left*), or SSSSSS sequences (*second left*), were significantly enriched to the upward (*P* < 1 × 10^−15^) by NCD38 treatment, but significantly downward (*P* < 1 × 10^−15^) by NCD38-β_2_P_4_ treatment. On the contrary, WWWWWW or 6-bp sequences including five W and one S (*second right*), or WWWWWW sequences (*the most right*), were further enriched to the upward (*P* < 1 × 10^−15^) by NCD38-β_2_P_4_ treatment, indicating that AT-rich regions were likely and GC-rich regions were unlikely activated by NCD38-β_2_P_4_.

We next investigated whether NCD38-β_2_P_4_ could preferentially target AT-rich regions. We analyzed the appearance of 6-bp DNA sequences within 250 bp from the center of the increased H3K27ac peaks. Strikingly, all the top 10 sequences were WWWWWW, whereas GC-rich sequences frequently appeared in the bottom 10 sequences (Figure 7A). Compared with NCD38 treatment, the appearance of SSSSSS sequences (where S indicates C or G) or 6-bp sequences including five S and one W significantly decreased in NCD38-β_2_P_4_ treatment (*P* < 1 × 10^−15^). On the contrary, the appearance of WWWWWW sequences or 6-bp sequences including five W and one S significantly increased (*P* < 1 × 10^−15^) (Figure 7B).

**Figure 8:**
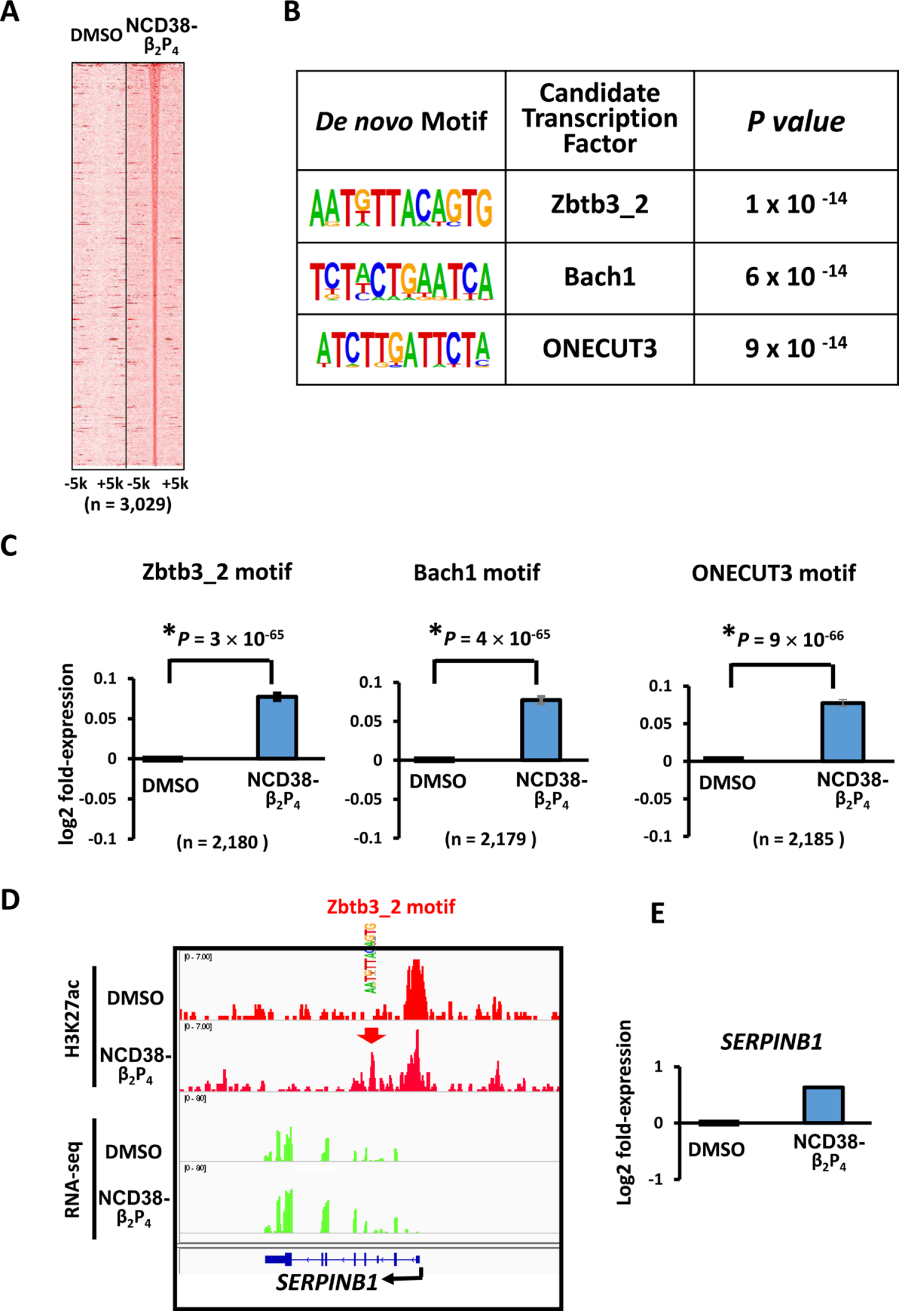
Enrichment of AT-rich motifs following NCD38-β_2_P_4_ treatment (**A**) Heat maps showing read densities of H3K27ac within ± 5 kb around H3K27ac-increased regions. (**B**) *De novo* motif analysis. The motives significantly enriched around H3K27ac-increased regions, Zbtb3_2, Bach1, and ONECUT3, were all AT-rich sequences. (**C**) Expression of genes with enriched motifs. Expression levels of genes nearest to the motives of Zbtb3_2, Bach1, and ONECUT3 with increased H3K27ac levels, were significantly upregulated by NCD38-β_2_P_4_. (**D**) Representative image of *SERPINB1*. H3K27ac level was increased at Zbtb3_2 motif site. (**E**) Representative RNA-seq results of *SERPINB1*. Expression of *SERPINB1* was increased.

*De novo* motif analysis revealed that the motifs significantly enriched around the increased H3K27ac peaks were Zbtb3_2, Bach1, and ONECUT3 (*P* = 1 × 10^−14^, *P* = 6 × 10^−14^, and *P* = 9 × 10^−14^, respectively), which were all AT-rich sequences (Figure 8A and 8B). Consistent with these findings, RNA-seq analysis revealed that the expression levels of genes nearest to these motifs with increased H3K27ac levels were significantly upregulated by NCD38-β_2_P_4_ (*P* = 3 × 10^−65^, *P* = 4 × 10^−65^, and *P* = 9 × 10^−66^, respectively; Figure 8C), e.g. *SERPINB1* activated by increase of H3K27ac at an enhancer with Zbtb3_2 motif (Figure 8D and 8E).

### ChIP-seq and RNA-seq analyses of NCD38-β_2_PIPP-treated cells

**Figure 9:**
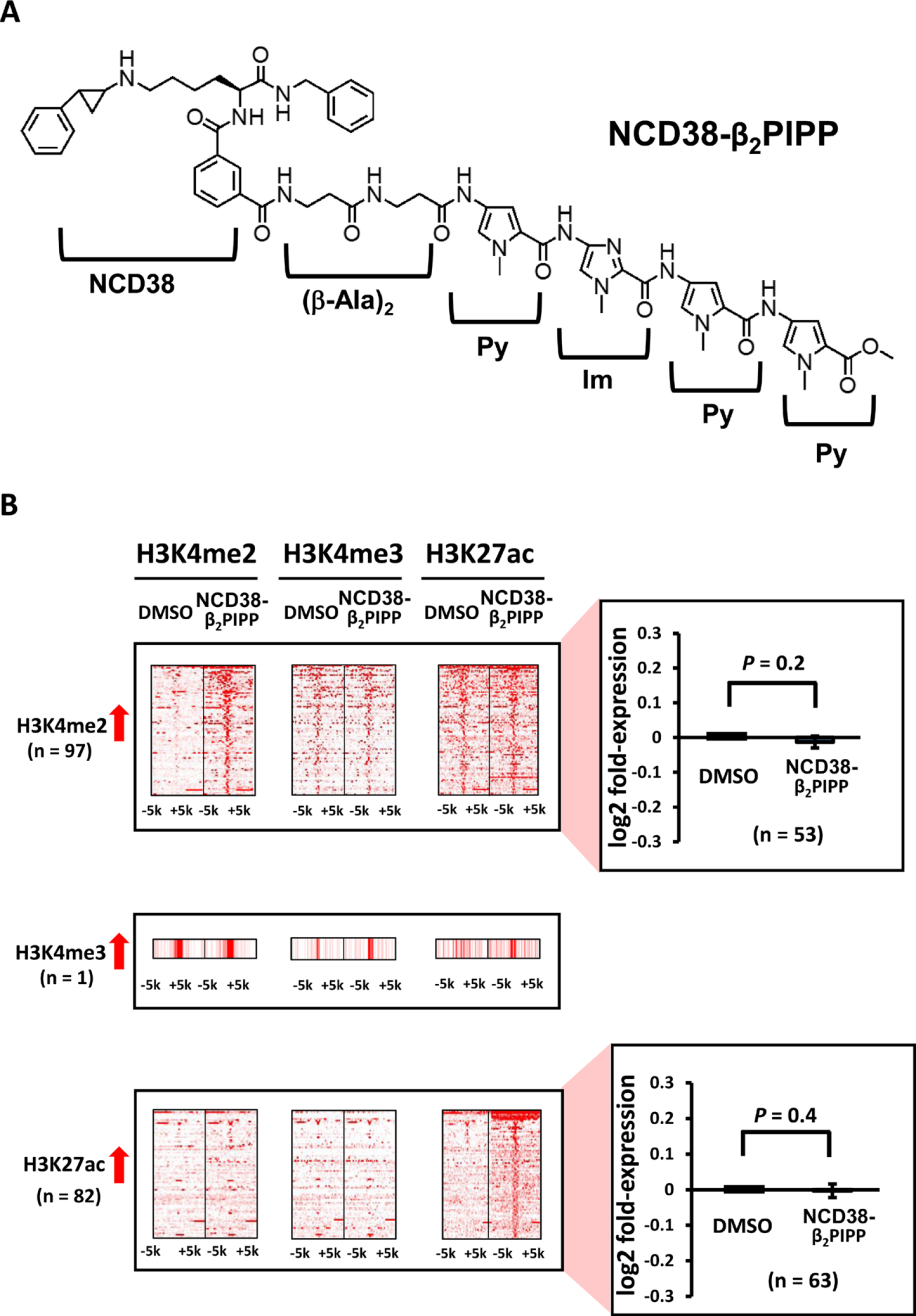
Alterations in histone modification by NCD38-β_2_PIPP treatment (**A**) Chemical structure of NCD38-β_2_PIPP. A Py-Im polyamide Py-Im-Py-Py, was conjugated with NCD38, with the dipeptide of β-alanine inserted. The conjugate recognizes WWCGWW sequences, and the specific binding to the sequence was confirmed (See Supplementary Figure 2). (**B**) Heat maps showing the read densities of ChIP-seq within ± 5 kb around the center position of ChIP-seq peaks. Less number of regions (97 and 82) showed more than 3-fold increases in H3K4me2 and H3K27ac levels, respectively, compared with treatment by parental NCD38 (*See* Figure 1B). Expression of genes nearest to these H3K4me2-increased and H3K27ac-increased peaks were not significantly up-regulated.

Next, another 4-mer polyamide Py-Im-Py-Py (PIPP) was conjugated with NCD38 with the dipeptide of β-alanine inserted as a linker, to develop NCD38-β-β-Py-Im-Py-Py (NCD38-β_2_PIPP) (Figure 9A). The cell permeability and nuclear localization of the Py-Im polyamide were confirmed using PIPP conjugated with FITC (Supplementary Figure 1). Specific recognition of WWCGWW sequence by NCD38-β_2_PIPP was confirmed; NCD38-β_2_PIPP selectively bound to oligo DNA with WWCGWW sequence, but not to that with GWCGWG, GWGCWG, WWWGCWWW, or WWWWWW (Supplementary Figure 2). The inhibitory activity of NCD38-β_2_PIPP against histone demethylase LSD1 was confirmed to be similar to the parental NCD38 (Supplementary Figure 3A). It was also confirmed that NCD38-β_2_PIPP lacked inhibitory activity against HDAC (Supplementary Figure 3B).

**Figure 10:**
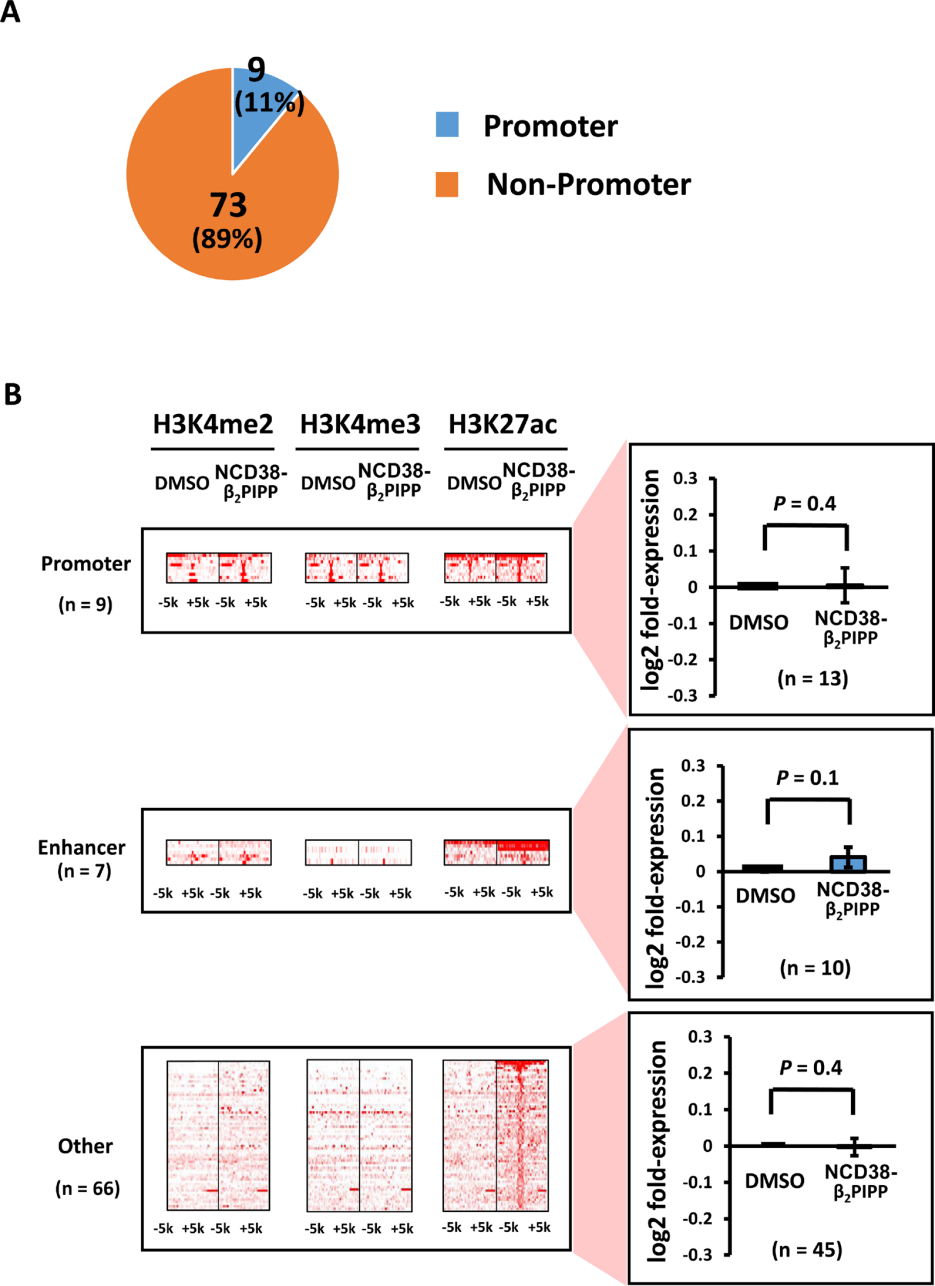
Distribution of the H3K27ac-increased regions following NCD38-β_2_PIPP treatment (**A**) Pie chart for the distribution of H3K27ac peaks. Where 9 of the 82 H3K27ac-increased regions (11%) were promoter regions, 73 regions (89%) were distributed in non-promoter regions. (**B**) Heat maps showing read densities of H3K4me2, H3K4me3, and H3K27ac within ± 5 kb around H3K27ac-increased regions. Among the 73 H3K27ac peaks in non-promoter regions, the majority was other regions.

When RKO cells were treated with NCD38-β_2_PIPP, fewer regions (97 and 82) showed more than 3-fold increases in H3K4me2 and H3K27ac levels compared with NCD38 treatment (Figure 9B). The majority (89%) of 82 H3K27ac-increased regions were distributed in non-promoter regions, including seven enhancer regions and 66 other regions (Figure 10A), and there was no overlap of activated regions between NCD38 and NCD38-β_2_PIPP treatment. The upregulation of genes nearest to the H3K27ac peaks at enhancer regions was not significantly observed (*P* = 0.1), perhaps due to small number of genes analyzed (*n* = 10) (Figure 10B).

**Figure 11:**
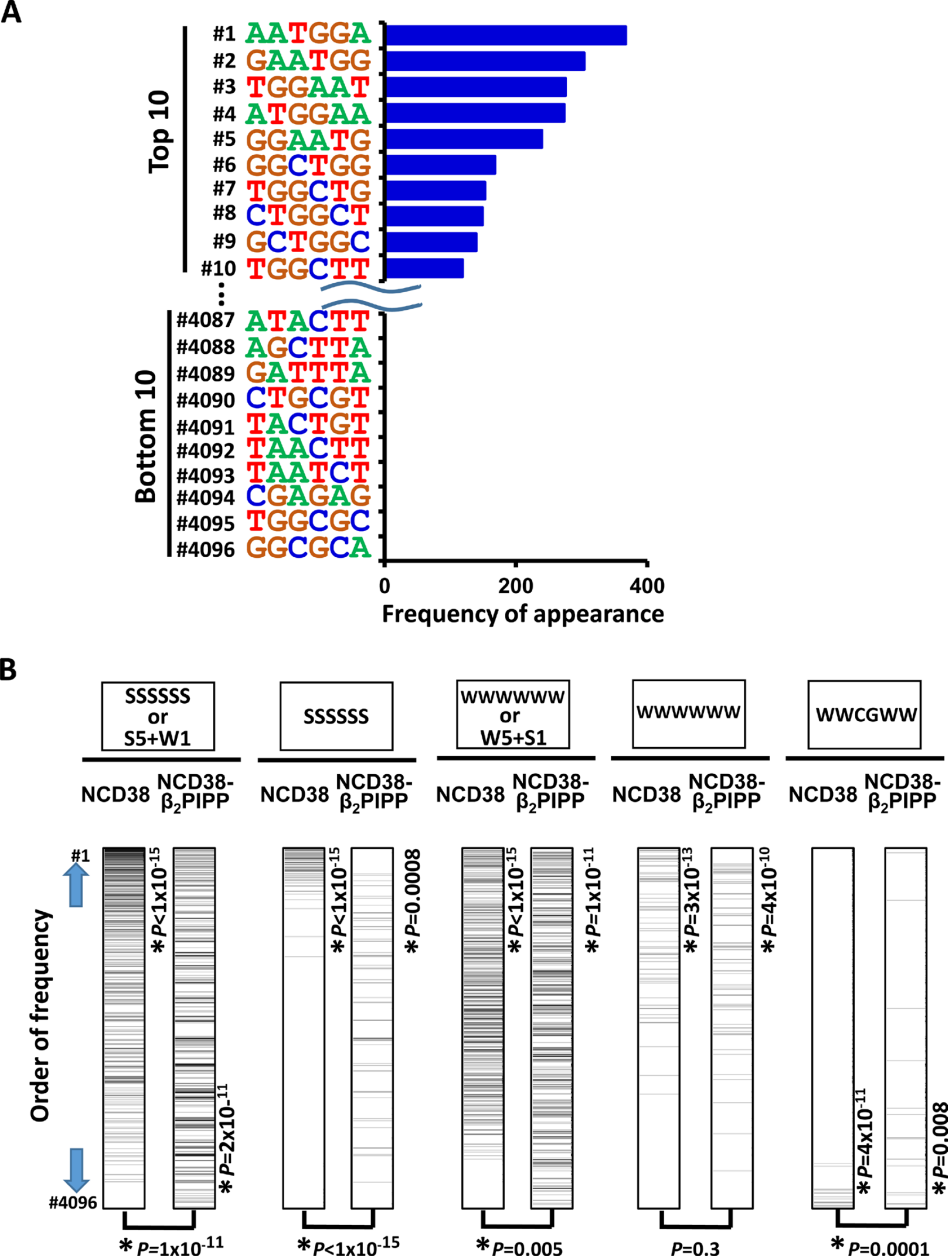
Appearance of DNA sequences in regions activated by NCD38-β_2_PIPP (**A**) Frequencies of appearance of 6-bp sequences within 250 bp from the center of the increased H3K27ac peaks. GC-contents of top 5 and top 10 sequences decreased to 40% ± 4% and 55% ± 6%, respectively (*See also* Figure 3B). (**B**) Significant activation of regions with WWCGWW sequences by NCD38-β_2_PIPP treatment. Total of 4,096 6-bp sequences were sorted by the order of frequency of appearance. SSSSSS or 6-bp sequences including five S and one W (*the most left*), or SSSSSS sequences (*second left*), were significantly enriched to the upward (*P* < 1 × 10^−15^) by NCD38 treatment, and these frequencies were significantly decreased (*P* = 1 × 10^−11^ or *P* < 1 × 10^−15^, respectively) by NCD38-β_2_PIPP treatment. WWWWWW or 6-bp sequences including five W and one S (*middle*), were enriched to the upward (*P* < 1 × 10^−15^) by NCD38-β_2_P_4_ treatment, and these frequencies were significantly decreased (*P* = 0.005) by NCD38-β_2_PIPP treatment. On the contrary, frequencies of WWCGWW sequences (*the most right*) were significantly increased (*P* = 0.0001) by NCD38-β_2_PIPP treatment, indicating that the former sequences were more unlikely, and WWCGWW sequences were more likely recognized by NCD38-β_2_PIPP.

To investigate whether NCD38-β_2_PIPP could preferentially target genomic regions containing WWCGWW, we analyzed the appearance of 6-bp DNA sequences within 250 bp from the center of the increased H3K27ac peaks in NCD38-β_2_PIPP treatment (Figure 11). GC-contents of top 5 and top 10 sequences were 40% ± 4% and 55% ± 6%, respectively (Figure 11A), which were significantly lower than those of top 5 and top 10 sequences in the treatment by NCD38 (Figure 3B). WWCGWW was not observed in top 10 sequences (Figure 11A), possibly because CG sequence is known to appear less frequently than expected, i.e. approximately 0.2-fold. The top rank of WWCGWW among 4,096 sequences, however, was markedly increased from #3,592 in NCD38 treatment to #41 in NCD38-β_2_PIPP treatment (Figure 11B, the most right).

Among 4,096 6-bp sequences, the appearance of SSSSSS sequences or 6-bp sequences including five S and one W significantly and remarkably decreased in NCD38-β_2_PIPP treatment (*P* = 1 × 10^−11^), compared with NCD38 treatment (Figure 11B). That of WWWWWW sequences or 6-bp sequences including five W and one S also decreased significantly but less remarkably in NCD38-β_2_PIPP treatment (*P* = 0.005). WWCGWW was the target sequence of NCD38-β_2_PIPP in binding dsDNA (Supplementary Figure 2) in 2:1 ligand to DNA stoichiometries, and the appearance of WWCGWW sequences significantly increased in NCD38-β_2_PIPP treatment (*P* = 0.0001) (Figure 11B). Meanwhile, WWWSWW could be presumably targeted if NCD38-β_2_PIPP binds dsDNA in 1:1 ligand to DNA stoichiometries, but the appearance of WWWSWW sequences did not increase in NCD38-β_2_PIPP treatment (*P* = 0.09) (Supplementary Figure 6). It is suggested that binding of NCD38-β_2_PIPP to dsDNA could be considered more likely in 2:1 ligand to DNA stoichiometries than 1:1.

RKO cells were treated with PIPP without conjugation of NCD38 for 30 days, and ChIP-seq analyses were performed. Among the 82 regions with >3-fold increase of H3K27ac levels after 30-day treatment with the conjugate, none of them showed >3-fold or >1.5-fold increase after treatment with PIPP (Supplementary Figures 7 and 8), suggesting that activation of regions should be due to the conjugated NCD38, not the effect of Py-Im polyamide itself.

### Inhibitor treatment for shorter period

While activation of these regions was observed in RKO cells treated with inhibitors for 30 days, these regions were not so activated in RKO cells treated for four days (Supplementary Figures 9–16). Among the 458 regions with >3-fold increase of H3K27ac levels after 30-day treatment with NCD38, none of them showed >3-fold increase after 4-day treatment; 11 regions with >1.5-fold increase and 447 with <1.5-fold increase. Gene activation was not observed after 4-day treatment, either (Supplementary Figures 9–11). Similarly, among the 234 regions with >3-fold increase of H3K27ac levels after 30-day treatment with NCD38-β_2_P_4_, none of them showed >3-fold increase after 4-day treatment, and no gene activation was observed (Supplementary Figures 12–14). Also the 82 regions with increase of H3K27ac levels after 30-day treatment with NCD38-β_2_PIPP, were not activated after 4-day treatment (Supplementary Figures 15 and 16).

## DISCUSSION

Although epigenetic aberrations play a significant role in carcinogenesis, inhibitors of epigenetic modifying enzymes are under development with the goal of establishing epigenetic therapy. Two DNMT inhibitors and four HDAC inhibitors have been approved by the FDA, and inhibitors of other epigenetic enzymes are being examined in clinical trials. These agents, however, reprogram the epigenome broadly and randomly; thus, unfavorable side effects can occur, along with antitumor effects [24, 25]. We therefore applied Py-Im polyamides to analyze whether epigenomic status could be altered in a sequence-specific manner by conjugating Py-Im polyamides with an LSD1 inhibitor, NCD38. NCD38-β_2_P_4_, recognizing WWWWWW sequence, and NCD38-β_2_PIPP, recognizing WWCGWW sequence, epigenetically altered regions frequently including the recognition sequences.

Py-Im polyamides have affinities for specific DNA sequences as strong as transcription factors, and these molecules can interfere with the binding of transcription factors to their recognition sites [26, 29, 31]. Applying the principles of these studies, we and Dervan's group recently demonstrated that Py-Im polyamides recognizing DNA with CpG sequences inhibited the induction of DNA methylation *in vitro* and *in cellulo* [35, 36]. While these studies showed that Py-Im polyamides could be utilized to inhibit DNA methylation in a region-selective manner, the present study suggested that Py-Im polyamides could be also utilized for region-selective alteration of histone modification by conjugation with epigenetic inhibitors.

Similar to our study, Sugiyama and colleagues conjugated a library of Py-Im polyamides with HDAC inhibitors or histone acetyl transferase activators and reported that each conjugate shows histone acetylation and gene activation in living cells in a different group of genes [37−39]. Because these conjugates are small molecules, they can easily be taken up through the cell membrane and localized to the nuclei [28, 36, 40]. In these previous reports, however, the link between epigenetically altered regions and sequences recognized by each Py-Im polyamide was not necessarily clear. In this study, we clearly demonstrated that NCD38-β_2_P_4_ and NCD38-β_2_PIPP preferentially modulated regions containing their recognizing sequences. Recently, Ansari and colleagues conjugated JQ1, a ligand of BRD4, with a Py-Im polyamide recognizing GAA repeats, and the conjugate successfully activated the repressive region with GAA repeats via recruitment of BRD4 [41].

NCD38 is an inhibitor of LSD1, a demethylase for H3K4me1 and H3K4me2, but not H3K4me3. NCD38 was previously shown to target LSD1 and increase H3K4me2 level [42], and the inhibition of LSD1 activity by NCD38 and its conjugates was also confirmed in this study (Supplementary Figure 3A). In RKO cells, H3K4me2 levels were increased in 103 regions by NCD38 treatment, accompanied by significant activation of nearby genes, whereas increased H3K4me3 levels were barely observed. Interestingly, a significant increase in H3K27ac levels was also observed in the present study, similar to a previous study of NCD38 treatment of acute myeloid leukemia cells [20]. The mechanism mediating the increase in H3K27ac levels following treatment with LSD1 inhibitor has not yet been fully clarified, and NCD38 and its conjugates did not directly inhibit HDAC activity (Supplementary Figure 3B). But it was suggested that formation of the Co-REST complex might be involved [20]. Co-REST was identified as a corepressor of the RE-1 silencing transcription factor REST [43]. LSD1 is a component of the repressor complex including HDAC1 or HDAC2, BRAF35, and Co-REST; LSD1 rapidly degrades in the absence of Co-REST [44–46]. Conditional deletion of LSD1 revealed a reduction in CoREST protein and HDAC activity, resulting in an increase of histone acetylation [47], and knockdown of LSD1 by siRNA also lead to an increase of histone acetylation [48]. The HDAC molecule in the complex may be simultaneously inhibited when LSD1 was inhibited during treatment with NCD38, causing H3K27ac levels to increase.

Moreover, LSD1 also works on demethylation of H3K9 residue and the association of LSD1 with the androgen receptor has been demonstrated to switch its substrate specificity from H3K4me/me2 to H3K9me/me2 to promote transcription of target genes [49, 50]. Gene regulation by NCD38 and its conjugates through alteration on H3K9 modification might also be interesting in specific cell types.

Recently, methods modifying the clustered regularly interspaced short palindromic repeats-Cas9 system were applied for region-selective editing of epigenomes [51–54]. The technique to increase the expression of a specific gene is to tether the dCas9-sgRNA complex to a transcriptional activator and program it to bind nearby the transcriptional start site of a gene of interest [55]. The catalytic domain of human acetyltransferase p300 was fused to the C-terminus of dCas9, which catalyzes histone H3 lysine 27 (H3K27) acetylation at loci up to thousands of base pairs from the sgRNA-specified locus and results in transcriptional activation of genes [56]. Although Cas9-based tools have been suggested to be useful for modifying particular epigenomic statuses, these tools are all macromolecules that are difficult for cells to spontaneously take up. In contrast, our strategy to employ Py-Im polyamides and their conjugates with small molecule epigenetic inhibitors could be developed as cell-permeable and nuclear-accessible machineries.

The conjugates used in this study, NCD38-β_2_P_4_ and NCD38-β_2_PIPP, recognized no longer than 6 bp, whereas more than 15 bp are considered necessary to target a unique region in the human genome. Long Py-Im polyamides, such as those targeting more than 20 bp could be developed [57]. Short polyamides, however, could induce effective modification of the epigenome by binding multiple genomic regions. Multiple enhancer regions with the same motif for transcription factors or repeat sequences, such as satellite DNA sites, may be effectively targeted by conjugates of short polyamides. Thus, we are currently investigating the most effective polyamide length using LSD1 inhibitors conjugated with Py-Im polyamides of various lengths in several types of cancer cells.

In summary, we conjugated NCD38 with two different Py-Im polyamides and analyzed the regions epigenetically altered by parental NCD38 and the two conjugates, NCD38-β_2_P_4_ and NCD38-β_2_PIPP. The altered regions showed significant enrichment of the sequences recognized by the two conjugates, suggesting that conjugation of Py-Im polyamides may be useful for region-selective alteration of epigenomic status in a sequence-specific manner.

## MATERIALS AND METHODS

### Cell culture

The human colorectal cancer cell line RKO was purchased from the American Type Culture Collection (Manassas, VA, USA). RKO cells were maintained in Eagle's minimum essential medium containing 10% heat-inactivated fetal bovine serum, 100 U/mL penicillin, and 100 μg/mL streptomycin and were grown in a humidified incubator at 37° C. Total RNA from RKO cells was extracted using a QIAamp DNA Micro Kit (Qiagen, Hilden, Germany) and RNAeasy (Qiagen).

### Inhibitor treatment

RKO cells were treated with 2 μM NCD38, NCD38-β_2_P_4_, or NCD38-β_2_PIPP containing 0.1% dimethylsulfoxide (DMSO) for 30 days, and none of these inhibitors were toxic to RKO cells at 2 μM. Medium was refreshed every 5 days, and 2 μM DMSO, NCD38, NCD38-β_2_P_4_, or NCD38-β_2_PIPP was added when the medium was refreshed, and cells were passaged when necessary. On day 30, treated cells were fixed with 1% formaldehyde for subsequent epigenomic analyses. RNA was also isolated from cells treated for 30 days for transcriptome analysis. RKO cells treated with 2 μM of P_4_ or PIPP were collected on day 30 for epigenomic analysis, and cells treated with 2 mM DMSO, NCD38, NCD38-β_2_P_4_, or NCD38-β_2_PIPP were also collected on day 4 for epigenomic and transcriptome analyses.

### RNA extraction, library construction, and RNA sequencing (RNA-seq) analysis

RNeasy Mini Kit (Qiagen) was used to extract RNA from the cells isolated on day 30 and day 4, following treatment with DNaseI (Qiagen). Library preparation for RNA-seq was performed using a TruSeq Stranded mRNA Sample Prep Kit (Illumina, San Diego, CA, USA) according to the manufacturer's protocols. The RNA-seq data were submitted to the NCBI BioSample database (http://www.ncbi.nlm.nih.gov/biosample), and the accession numbers are GSM2894015 - GSM2894018 (day 30), and GSM3039396 - GSM3039399 (day 4). TopHat was used to align sequenced reads from the RNA-seq experiment, and Cufflinks was used for transcript assembly. Gene expression levels were represented as fragments per kilobase of exon per million mapped sequence reads.

### Chromatin immunoprecipitation (ChIP) and library construction

RKO cells were crosslinked with 1% formaldehyde for 10 min at room temperature. To obtain 0.125 M as a final concentration, 2.5 M glycine was added to 1% formaldehyde. An ultrasonic disrupter (BRANSON Digital Sonifier, Branson, Danbury, CT, USA) was used to sonicate the crosslinked chromatin to a size of 0.2–1 kb. About 2–5 μg antibody and 20 μL Protein G sepharose beads were mixed in IP dilution buffer and incubated for approximately 6 h at 4° C. Anti-H3K4me2 (#05-1334, Merck Millipore, Billerica, MA, USA), anti-H3K4me3 (#ab7766, Abcam, Cambridge, UK), and anti-H3K27ac (#39159, Active Motif, Carlsbad, CA, USA) antibodies were used in this study. Antibody-bound beads were washed with IP dilution buffer and added to the sonicated-chromatin sample. The mixture was incubated overnight at 4° C. After washing the beads, chromatin was eluted, followed by reverse crosslinking. Then, DNA purification was performed using QIAquick PCR purification kit (Qiagen) according to manufacturer's instruction. ChIP libraries were constructed using NEBNext ChIP-seq Library Prep Reagent Set for Illumina (NEB, Ipswich, MA, USA) according to the manufacturer's instructions. A Bioanalyzer (Agilent, Santa Clara, CA, USA) was used to quantify ChIP seq libraries.

### ChIP sequencing (ChIP-seq) analysis

Quantified ChIP-seq libraries were sequenced at a concentration of 4 pM on an Illumina Hiseq (Illumina). These ChIP-seq data were submitted to the NCBI BioSample database (http://www.ncbi.nlm.nih.gov/biosample), and the accession numbers are GSM2894019 - GSM2894030 (day 30), and GSM3039400 - GSM3039411 (day 4). The UCSC human genome (hg19) was used to map the sequenced reads in ChIP experiments. HOMER software (http://homer.salk.edu/homer/index.html) was used for peak detection. Annotation to the nearest gene was performed using GREAT (http://bejerano.stanford.edu/great/public/html/index.php). HOMER and TreeView were used to produce heat maps for calculating enrichment and visualization.

### Synthesis of PIPs and their conjugates

All reactants or reagents including dry solvents were obtained from commercial suppliers and used as received. Parental units of Py-Im polyamides were synthesized according to previously described methods [27, 58, 59]. NCD38 and its derivative were obtained by previously described methods [16, 17]. As shown in Supplementary Figure 17, positive-ion mass spectra were recorded by electrospray ionization (ESI-TOF). The molecular weight of NCD38-β_2_P_4_, C_60_H_70_N_13_O_10_^+^ [M^+^H], was calculated to be 1144.5363, and found to be 1144.5350. The molecular weight of NCD38-β_2_PIPP, C_60_H_69_N_14_O_10_^+^ [M^+^H], was calculated to be 1145.5316, and found to be 1145.5296. Detailed procedures and information for conjugation of Py-Im polyamides with NCD38 will be provided upon request.

### Distribution of fluorescein isothiocyanate (FITC)-labeled Py-Im polyamides in cultured RKO cells

RKO cells were seeded at a density of 1.0 × 10^5^ cells per 35-mm dish and grown in 2 mL medium. After 24 h, the living cells were incubated with 500 nM of FITC-conjugated P_4_ or PIPP containing 0.1% DMSO for 3 h. After the incubation, the cells were fixed with 1% HCHO for 2 h on ice. Fixed cells were washed twice with PBS followed by nuclear staining with 1.0 μg/mL 4’,6-diamidino-2-phenylindole. Cells were visualized with a BZ-X710 fluorescence microscope (KEYENCE, Osaka, Japan).

### LSD1 and HDAC assay *in vitro*

LSD1 and HDAC activities were assessed using an LSD1 fluorometric drug discovery kit (Enzo Life Sciences, New York, NY, USA) and Histone Deacetylase Activity Assay Kit (Abcam, Cambridge, UK) according to the manufacturer's instructions. Ten microliters of reaction mixture containing LSD1 (5 ng/mL), H3K4Me2 peptide-fragments (20 μM), CELLestial Red (1×), horseradish peroxidase (1×), and NCD38-conjugates (1–100 μM with 1% DMSO) were incubated at room temperature for 30 min. To assess HDAC inhibition, 10 μL of reaction mixture containing 1 μL of crude HDAC, Fluoro-Substrate Peptide (20 μM), HDAC assay buffer (1×), and NCD38-conjugates (10 μM with 1% DMSO) were incubated at room temperature for 20 min followed by addition of 4 μL Stop Solution and 1 μL Developer. Relative fluorescence units at 590 nm for LSD1 and 460 nm for HDAC were detected using a NanoDrop 3300 (Thermo Fisher Scientific, Waltham, MA, USA) with a white-light and UV-light source, respectively.

### Electrophoretic mobility shift assay (EMSA)

The 16- or 18-bp dsDNA containing targeting sequences for NCD38-β_2_P_4_ and NCD38-β_2_PIPP was prepared by annealing of oligo DNA described in Supplementary Table 1. In a 1.5 mL tube, 1.0 μM FAM-labeled dsDNA was incubated in 10 μL reaction solution containing the compounds, 10 mM Tris-HCl (pH 8.0) and 1% DMSO for 1 h at room temperature. The resultant complexes were loaded onto a 10% polyacrylamide gel and separated using EMSA in 0.5 × TBE. Selective binding between each conjugates and dsDNA was visualized using an LAS-3000 imaging system (Fujifilm, Tokyo, Japan)

### Bioinformatic and statistical analyses

Gene ontology enrichment was performed using GREAT (http://bejerano.stanford.edu/great/public/html/index.php). Enrichment of *de novo* motifs was performed using HOMER software (http://homer.salk.edu/homer/index.html). Gene expression levels and frequencies of particular sequences were compared using Student's *t*-tests.

## SUPPLEMENTARY MATERIALS FIGURES AND TABLE



## Abbreviations

LSD1: lysine-specific demethylase-1
Py: pyrrole
Im: imidazole
P_4_: Py-Py-Py-Py
PIPP: Py-Im-Py-Py
β-Ala: β
FAD: flavin adenine dinucleotide
FDA: US Food and Drug Administration
DNMT: DNA methyltransferase
HDAC: histone deacetylase
RNA-seq: RNA sequencing
ChIP: chromatin immunoprecipitation
ChIP-seq: ChIP sequencing
FITC: fluorescein isothiocyanate.
