# Supplementary material for: Region-specific alteration of histone modification by LSD1 inhibitor conjugated with pyrrole-imidazole polyamide
Source: Oncotarget. 2018 Jun 29;9(50):29316–35. doi: 10.18632/oncotarget.25451 (PMC6047668; doi:10.18632/oncotarget.25451)
Supplement: Supplementary file 1 [file oncotarget-09-29316-s001.pdf]

Region-specific alteration of histone modification by LSD1 inhibitor conjugated with pyrrole-imidazole polyamide

SUPPLEMENTARY MATERIALS

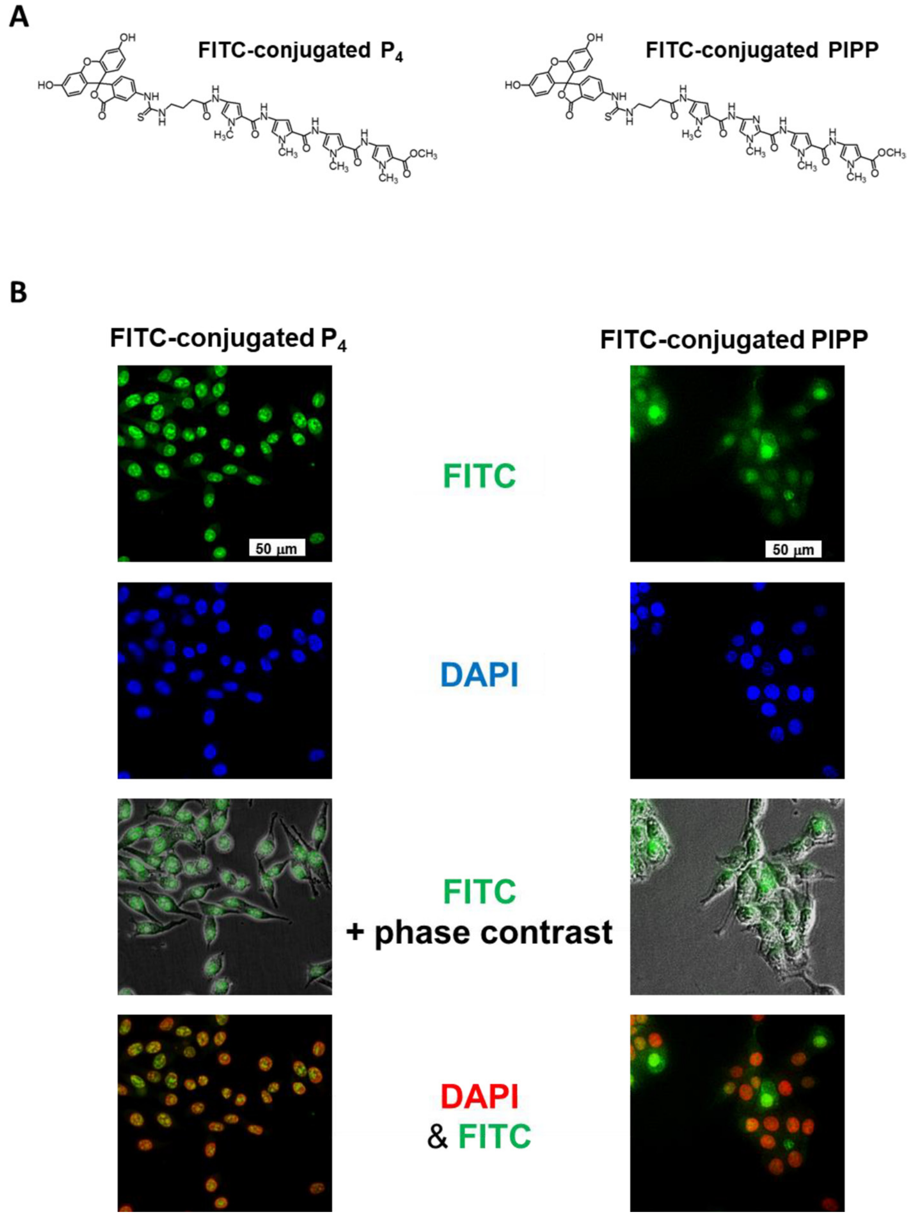

Supplementary Figure 1: Distribution of FITC-conjugated Py-Im polyamides in living RKO cells after 3 h incubation. (**A**) Chemical structures of FITC-conjugated P_4 and PIPP. (**B**) Living RKO cells were incubated with 500 nM of FITC-conjugated Py-Im polyamides. After the incubation, cells were fixed with 1% formaldehyde. The cells were visualized by fluorescence microscopy following to nucleic staining with DAPI (*blue* or *red*). Scale bar indicates 50 μm. It is clearly indicated that FITC-conjugated Py-Im polyamides (*green*) were localized in the nuclei of RKO cells.

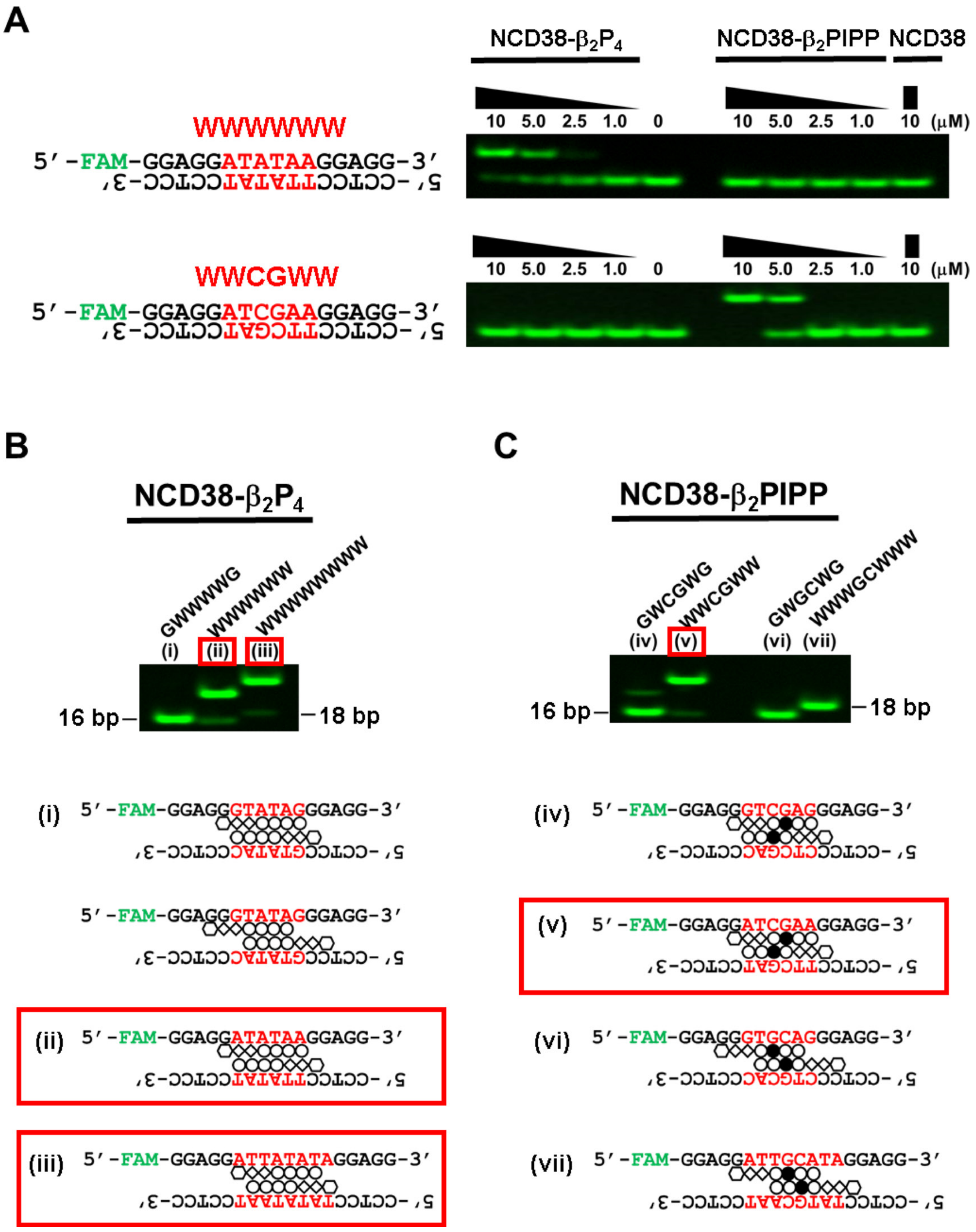

Supplementary Figure 2: EMSA analysis for NCD38-β_2P_4 and NCD38-β_2PIPP on a 10% polyacrylamide gel. (**A**) EMSA analysis was performed for double strand DNA (dsDNA) in presence NCD38-β_2P_4 (lanes 1-4), vehicle control (lane 5: 1% DMSO), NCD38-β_2PIPP (lanes 6-9), NCD38 (lane 10). It was demonstrated that NCD38-β_2P_4 bound to dsDNA containing WWWWWW, and NCD38-β_2PIPP to dsDNA containing WWCGWW. (**B**) EMSA analysis for dsDNAs containing 4-8 bp AT-rich sequences. *Open circle*, Py. *Closed circle*, Im. *Diamond*, β. *Hexagon*, NCD38. NCD38-β_2P_4 was shown to likely bind dsDNA containing WWWWWW (*red rectangle*). (**C**) EMSA analysis for dsDNAs containing CpG or GpC within 4-8 bp sequences. NCD38-β_2PIPP was shown to likely bind dsDNA containing WWCGWW (*red rectangle*).

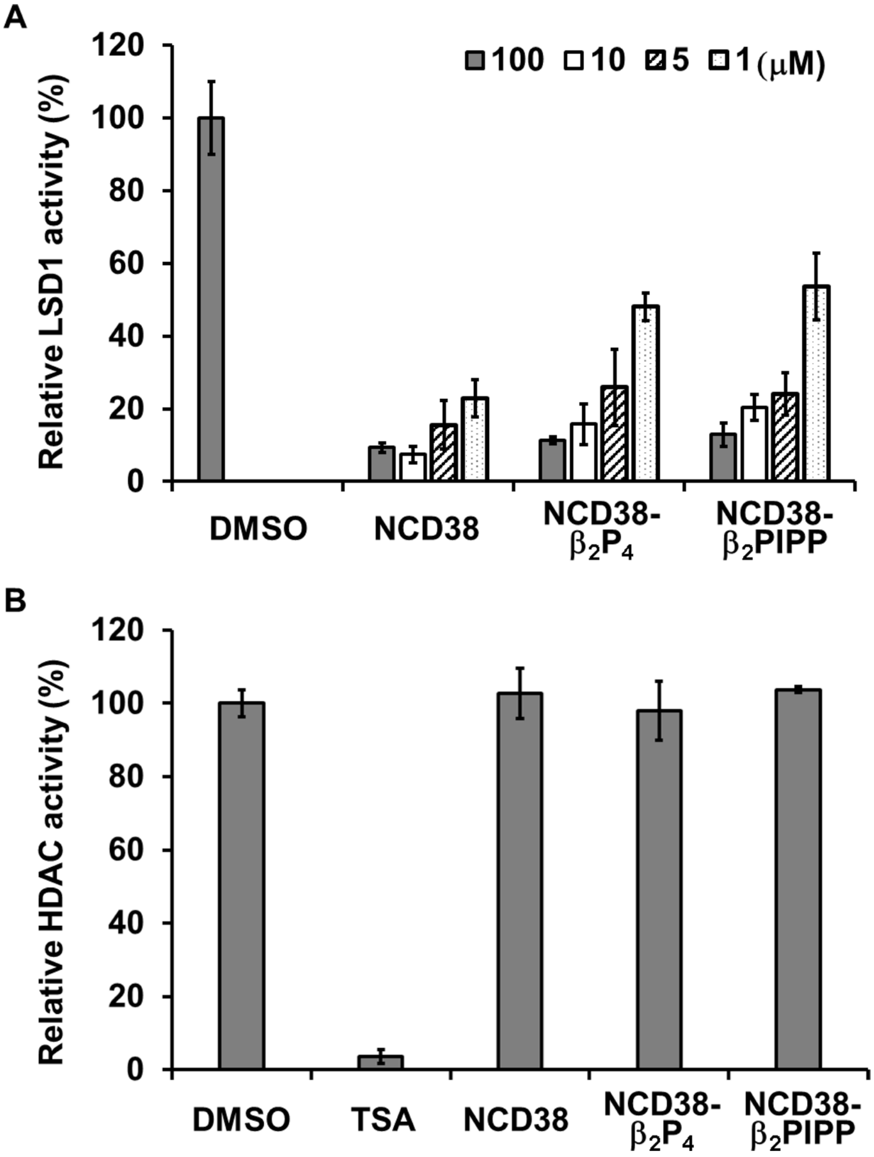

Supplementary Figure 3: Inhibition of LSD1 and HDAC activity. (**A**) Inhibition of LSD1 activity by the compounds *in vitro*. The LSD1 activity was assessed with LSD1 fluorometric drug discovery kit. LSD1 activities are shown as bars filled with *grey, light grey, slashed lines and bots* in presence 100, 10, 5 and 1 μM of the compounds, respectively. (**B**) Inhibition of HDAC activity by the compounds *in vitro*. The HDAC activity was assessed with HDAC Activity Assay Kit in presence 10 μM of the compounds. Trichostatin A (TSA) was used as positive control for HDAC inhibitor. Both LSD1 and HDAC activities were estimated by comparison with 1% DMSO control as 100%. *Error bars*, standard deviation of the means of triplicate samples. It was implied that activities of LSD1 inhibition by NCD38-β_2P_4 and NCD38-β_2PIPP were levels similar to that of parental NCD38, while no inhibition of HDAC was observed by treatment of NCD38, NCD38-β_2P_4 and NCD38-β_2PIPP.

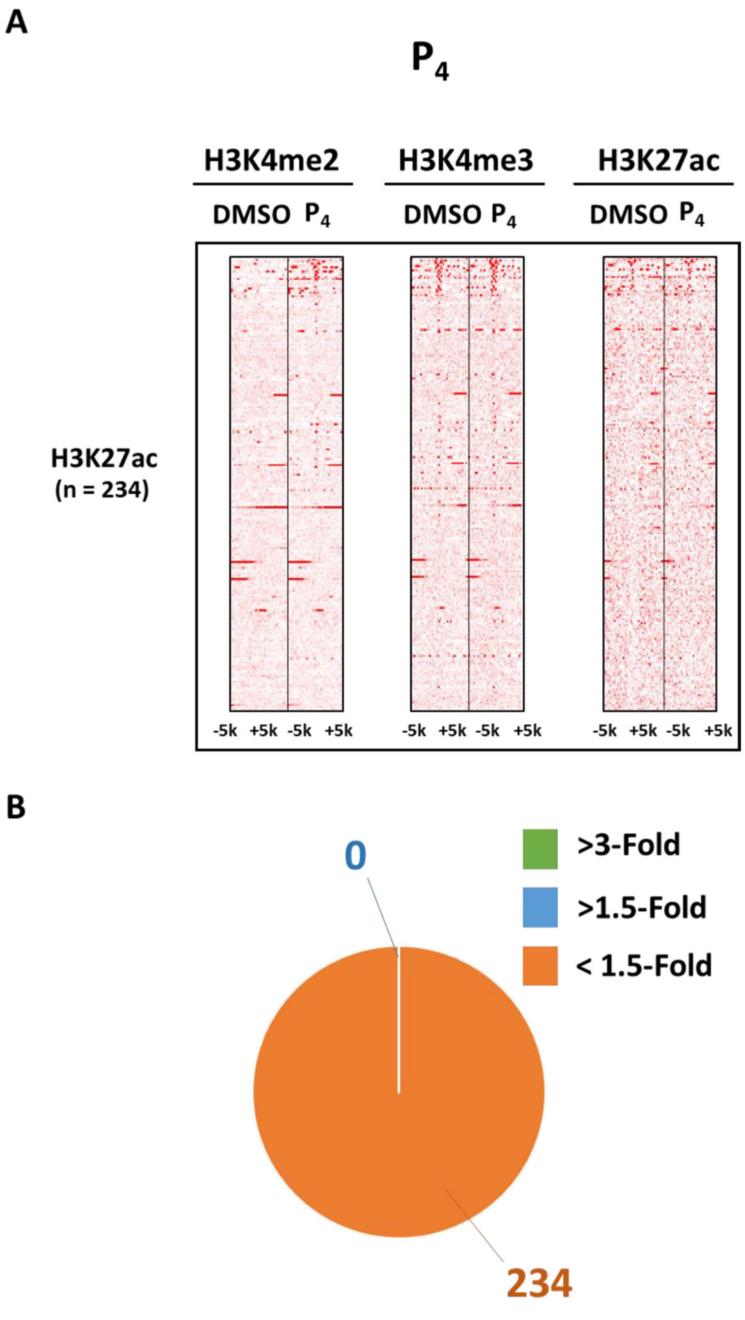

Supplementary Figure 4: Alteration of histone modification by P_4 treatment. Heatmap showed the regions where increase of H3K27ac levels was observed by treatment with NCD38-β_2P_4 (Figure 5B). The alteration was not observed by treatment with the Py-Im polyamide.

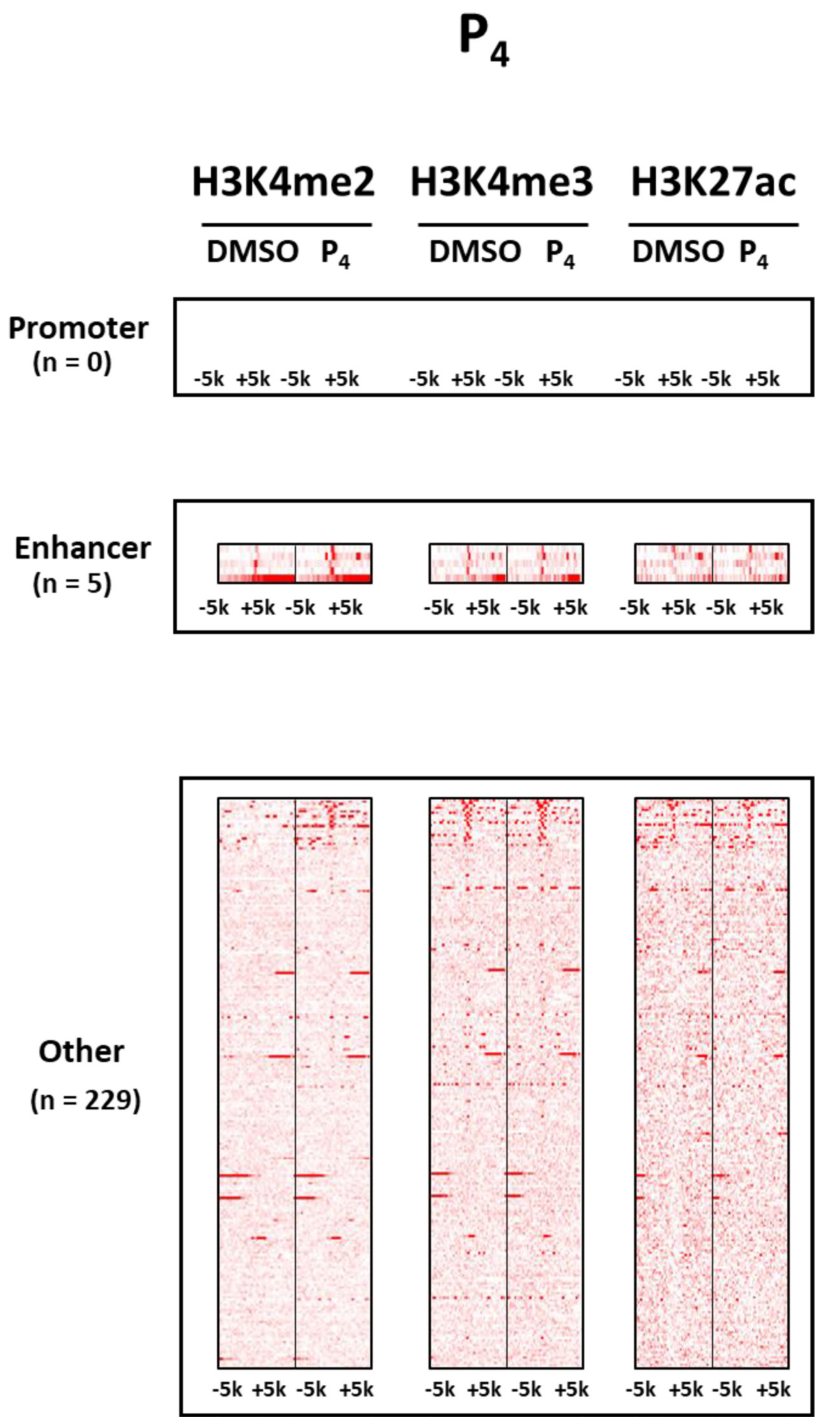

Supplementary Figure 5: Alteration of H3K27ac by P_4 treatment. Heatmap showed H3K27ac-increased regions in promoter, enhancer, and other regions observed by treatment with NCD38-β_2P_4 (Figure 6B). The alteration was not observed by treatment with Py-Im polyamide.

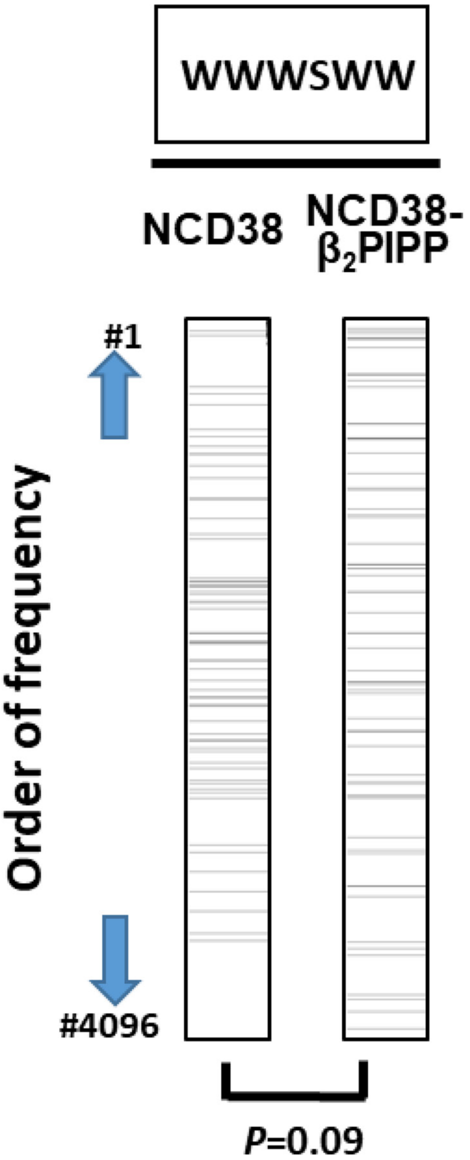

Supplementary Figure 6: Appearance of WWWSWW sequence. Total of 4,096 6-bp sequences were sorted by the order of frequency of appearance. WWWSWW could be presumably targeted if NCD38-β_2PIPP binds dsDNA in 1:1 ligand to DNA stoichiometries, but the appearance of WWWSWW sequences did not significantly increase in NCD38-β_2PIPP treatment (P = 0.09), compared with NCD38 treatment. Meanwhile, WWCGWW, target sequences of NCD38-β_2PIPP in 2:1 ligand to DNA stoichiometries (*See* Supplementary Figure 2), was significantly increased in NCD38-β_2PIPP treatment (*See* Figure 11). It is suggested that binding of NCD38-β_2PIPP to dsDNA could be considered more likely in 2:1 ligand to DNA stoichiometries than 1:1.

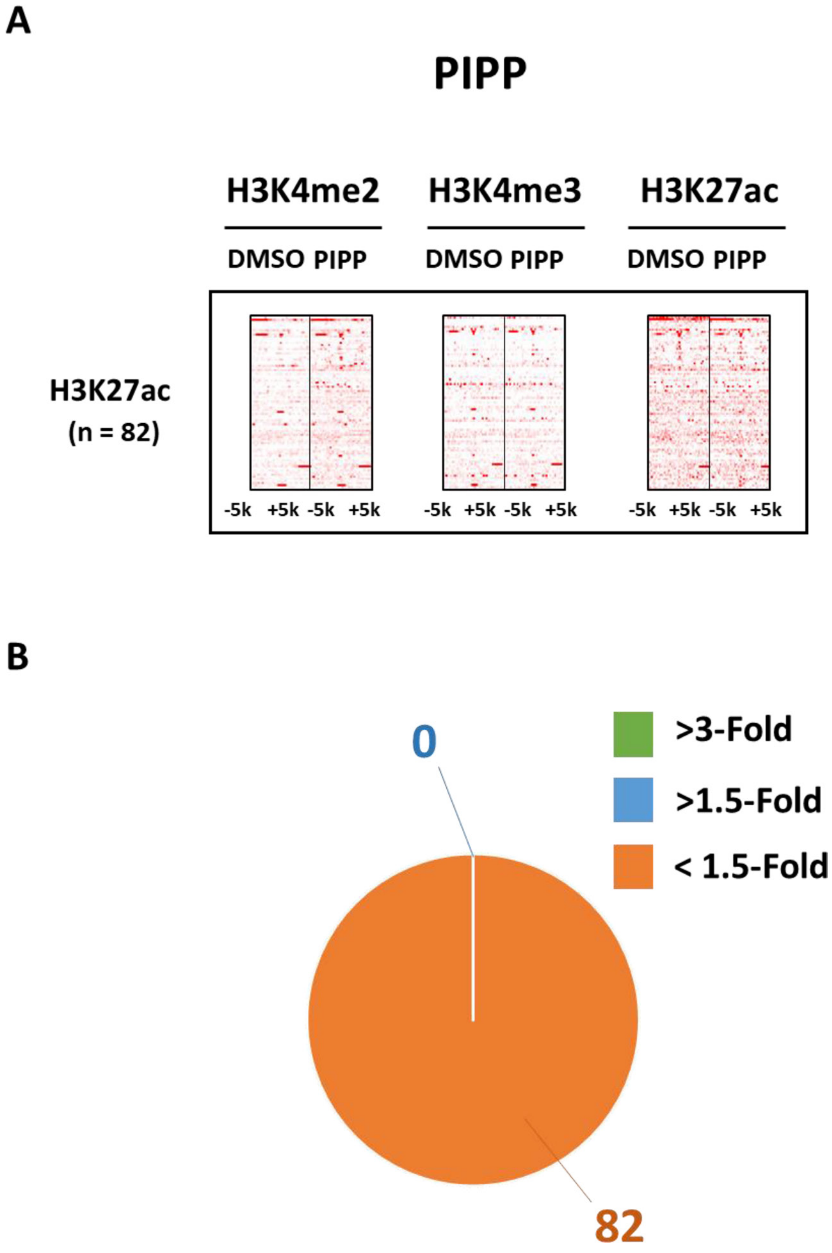

Supplementary Figure 7: Alteration of histone modification by PIPP treatment. Heatmap showed the regions where increase of H3K27ac levels was observed by treatment with NCD38-β_2PIPP (Figure 9B). The alteration was not observed by treatment with the Py-Im polyamide.

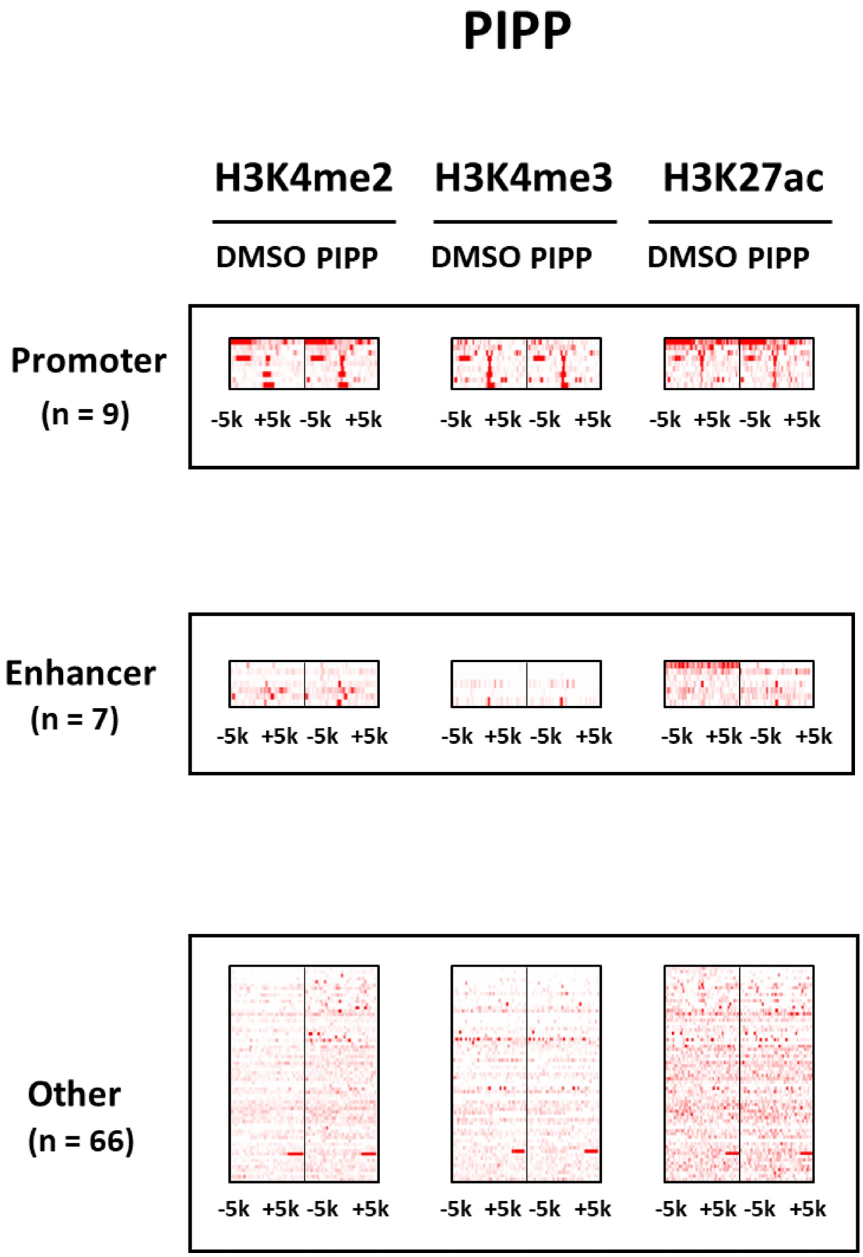

Supplementary Figure 8: Alteration of H3K27ac by PIPP treatment. Heatmap showed H3K27ac-increased regions in promoter, enhancer, and other regions observed by treatment with NCD38-β_2PIPP (Figure 10B). The alteration was not observed by treatment with the Py-Im polyamide.

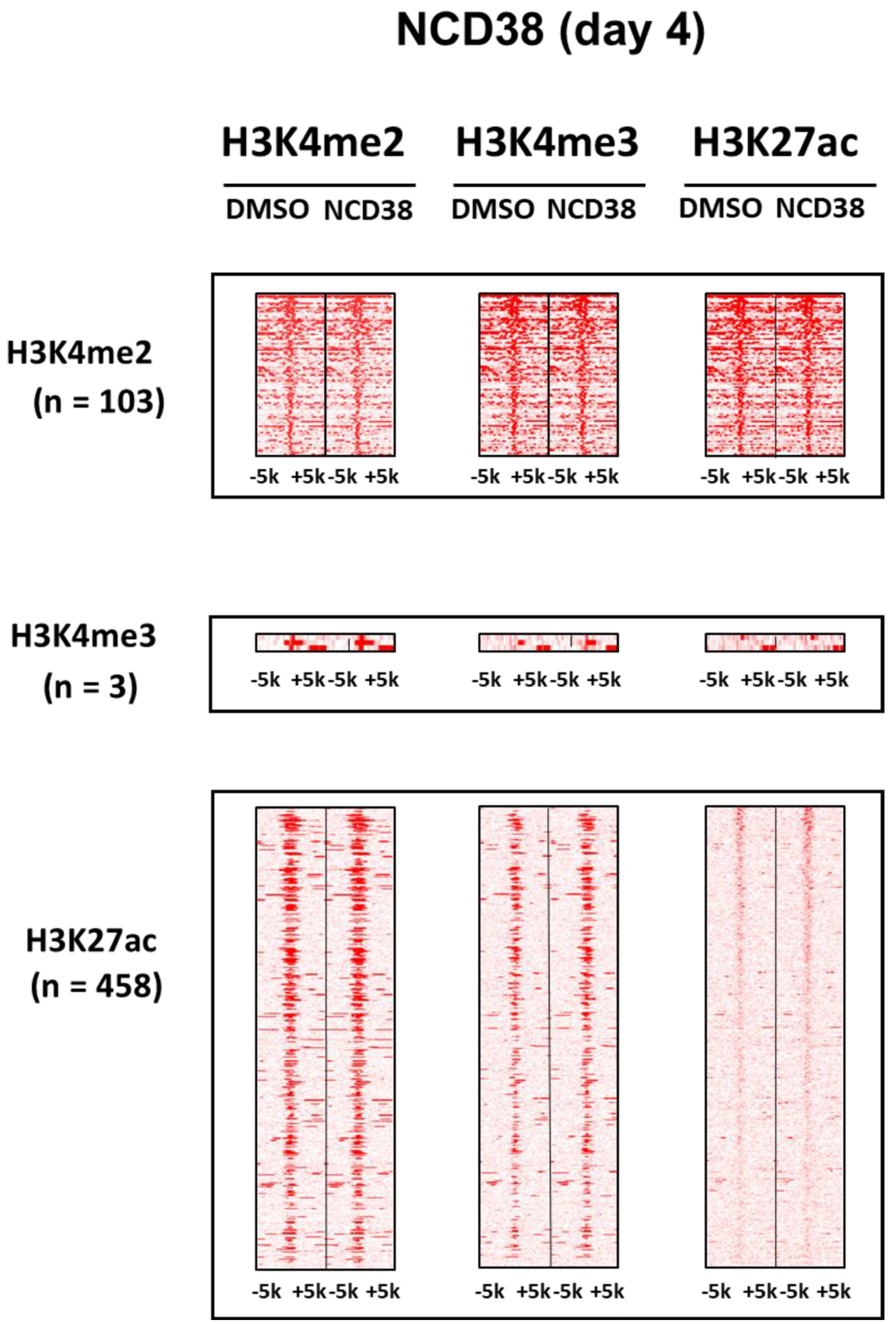

Supplementary Figure 9: Alteration of histone modification by NCD38 treatment for four days. Heatmap showed the regions where increase of histone modification levels was observed on day 30 (Figure 1B). The alteration was not so observed on day 4.

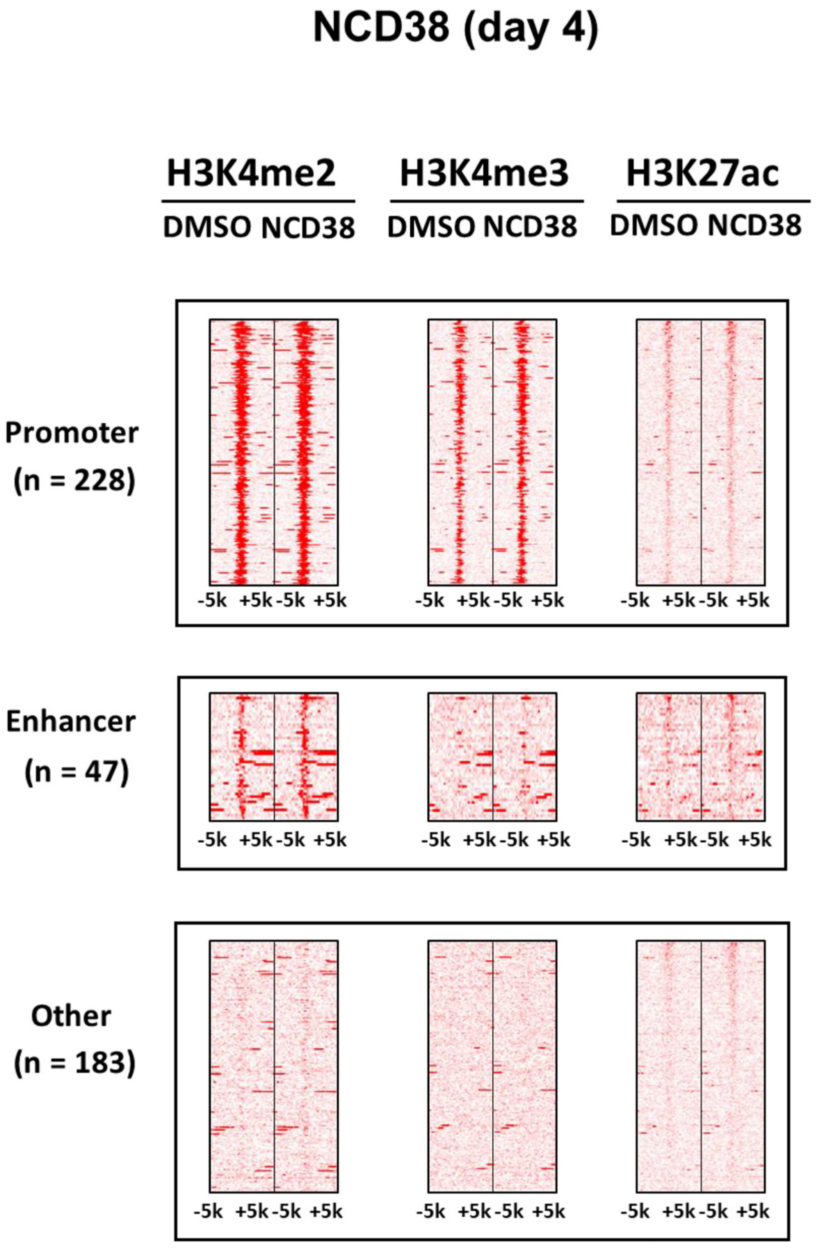

Supplementary Figure 10: Alteration of H3K27ac by NCD38 treatment for four days. Heatmap showed H3K27ac-increased regions in promoter, enhancer, and other regions observed on day 30 (Figure 2B). The alteration was not so observed on day 4.

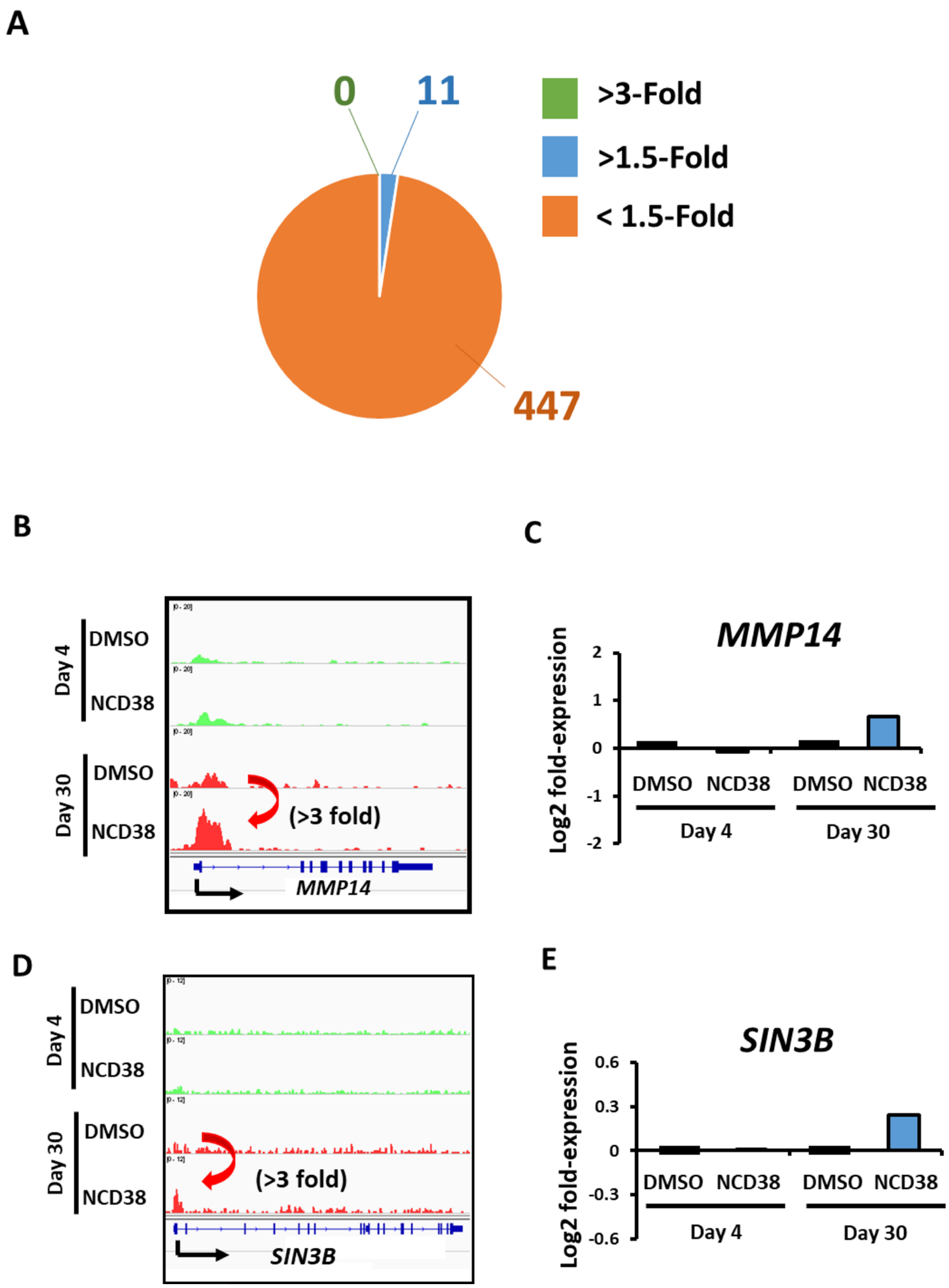

Supplementary Figure 11: Gene activation by NCD38 treatment for four days. (**A**) Among the 458 regions with >3-fold increase of H3K27ac levels on day 30, none of them showed >3-fold increase on day 4; 11 regions with >1.5-fold increase and 447 with <1.5-fold increase. A representative gene with >1.5-fold increase of H3K27ac (**B, C**) and one with <1.5-fold increase (**D, E**) were shown. Gene activation was not observed after 4-day treatment.

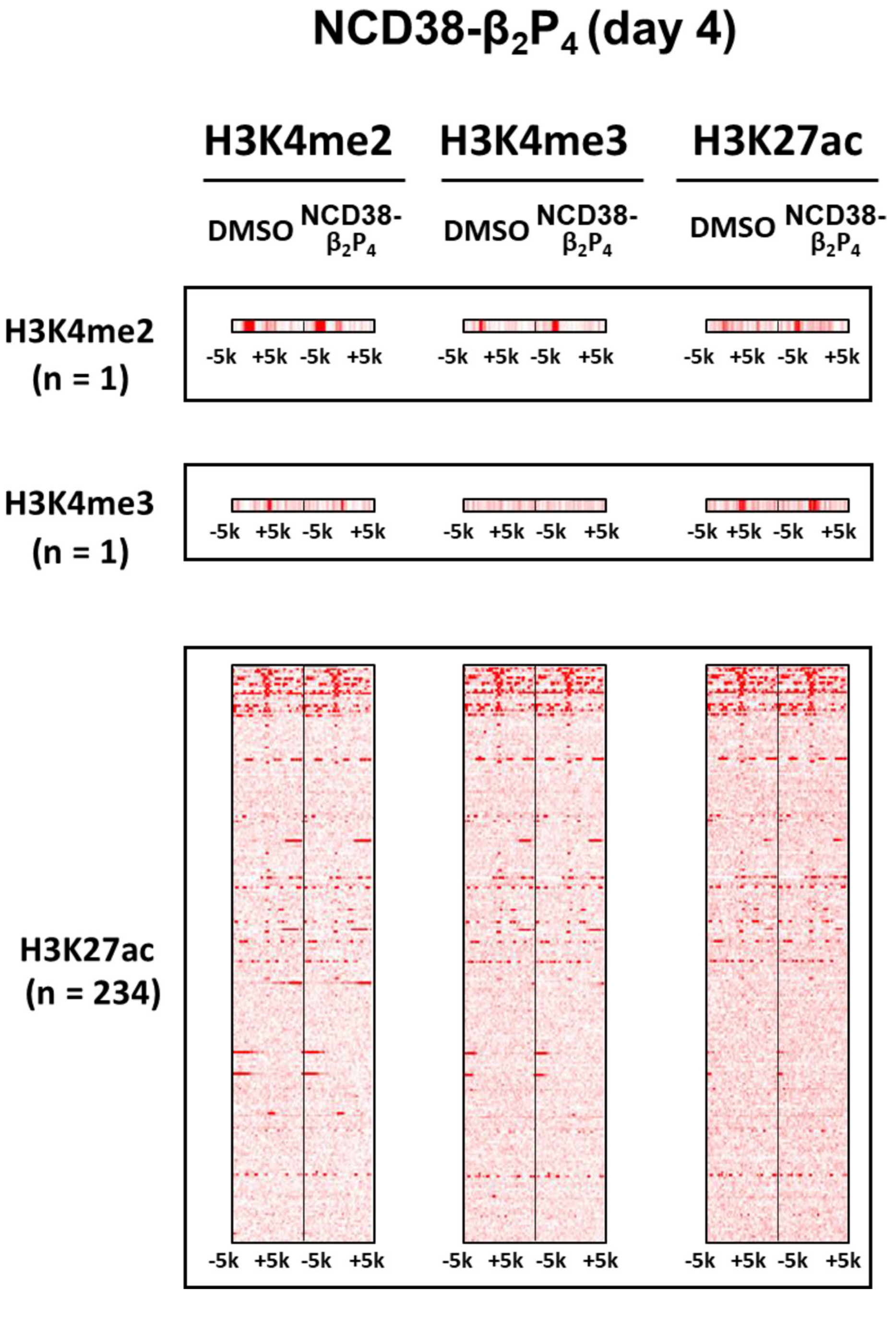

Supplementary Figure 12: Alteration of histone modification by NCD38-β_2P_4 treatment for four days. Heatmap showed the regions where increase of histone modification levels was observed on day 30 (Figure 5B). The alteration was not so observed on day 4.

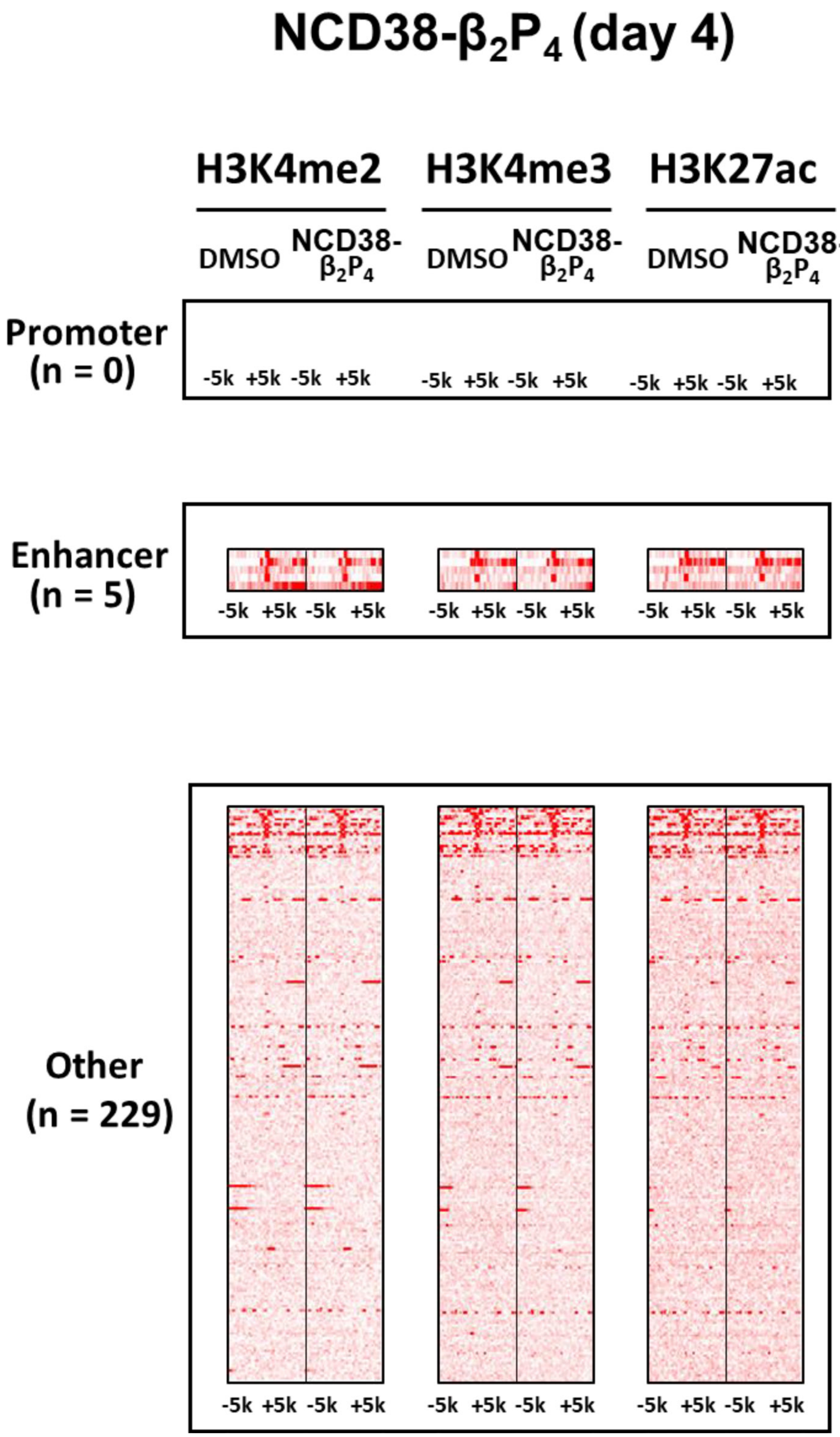

Supplementary Figure 13: Alteration of H3K27ac by NCD38-β_2P_4 treatment for four days. Heatmap showed H3K27ac-increased regions in promoter, enhancer, and other regions observed on day 30 (Figure 6B). The alteration was not so observed on day 4.

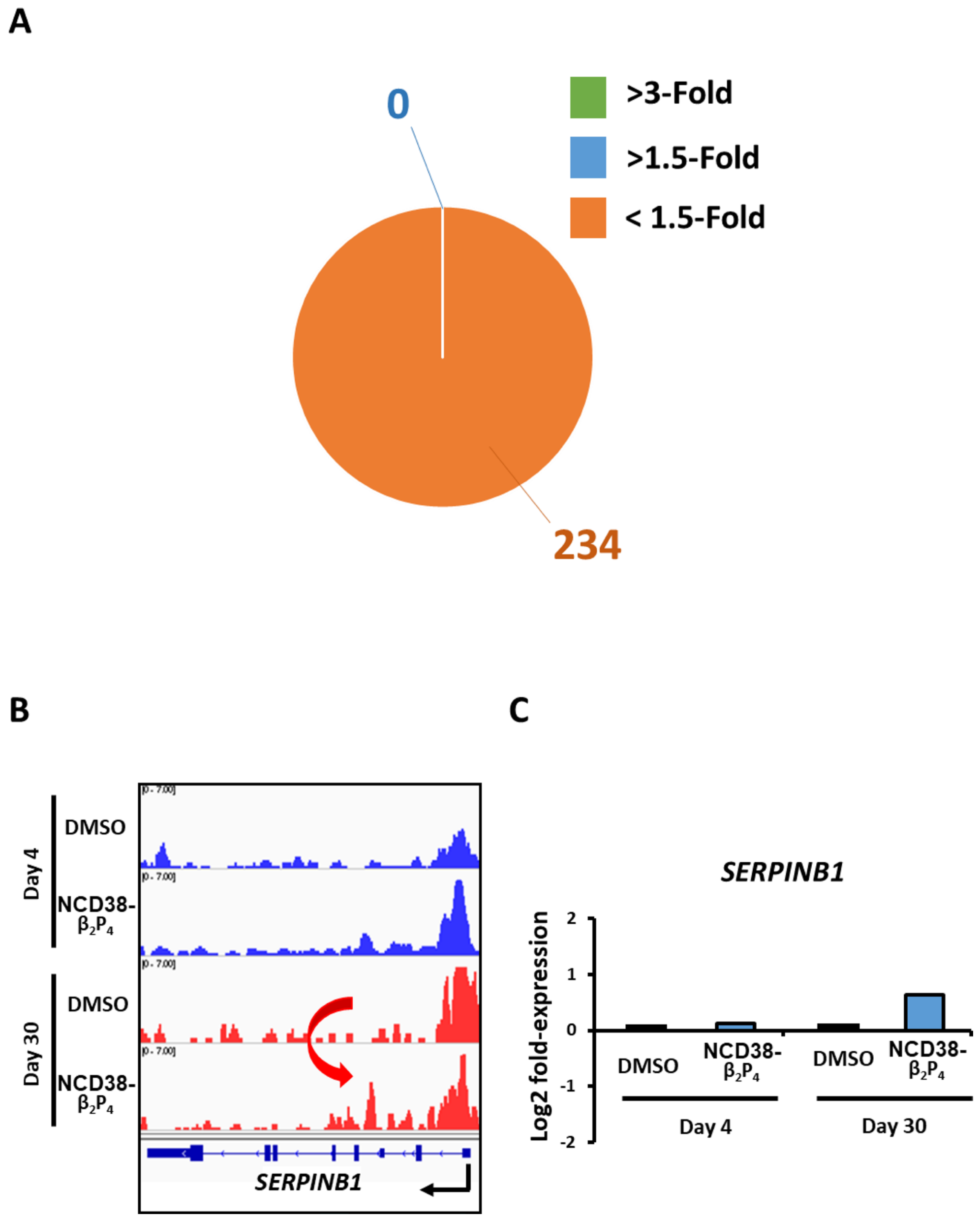

Supplementary Figure 14: Gene activation by NCD38-β_2P_4 treatment for four days. (**A**) Among the 234 regions with >3-fold increase of H3K27ac levels on day 30, none of them showed >3-fold increase or >1.5-fold increase on day 4. (**B, C**) A representative gene with <1.5-fold increase on day 4. Gene activation was not observed after 4-day treatment.

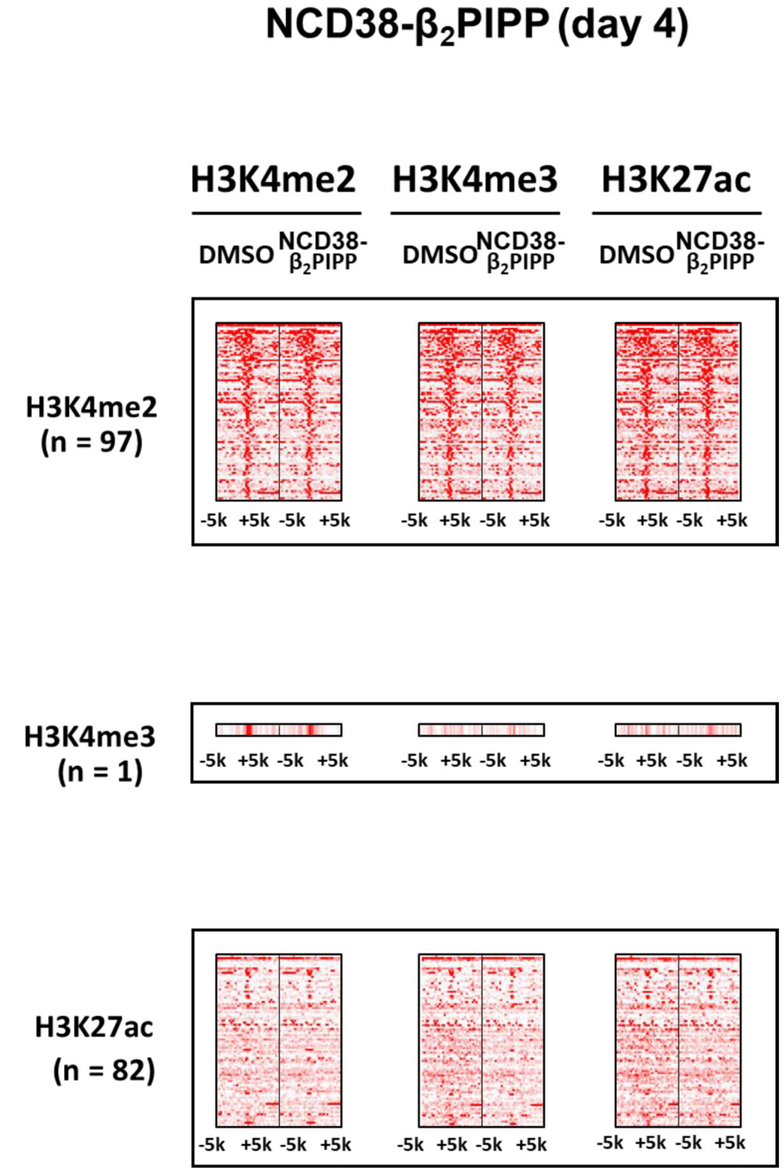

Supplementary Figure 15: Alteration of histone modification by NCD38-β_2PIPP treatment for four days. Heatmap showed the regions where increase of histone modification levels was observed on day 30 (Figure 9B). The alteration was not so observed on day 4.

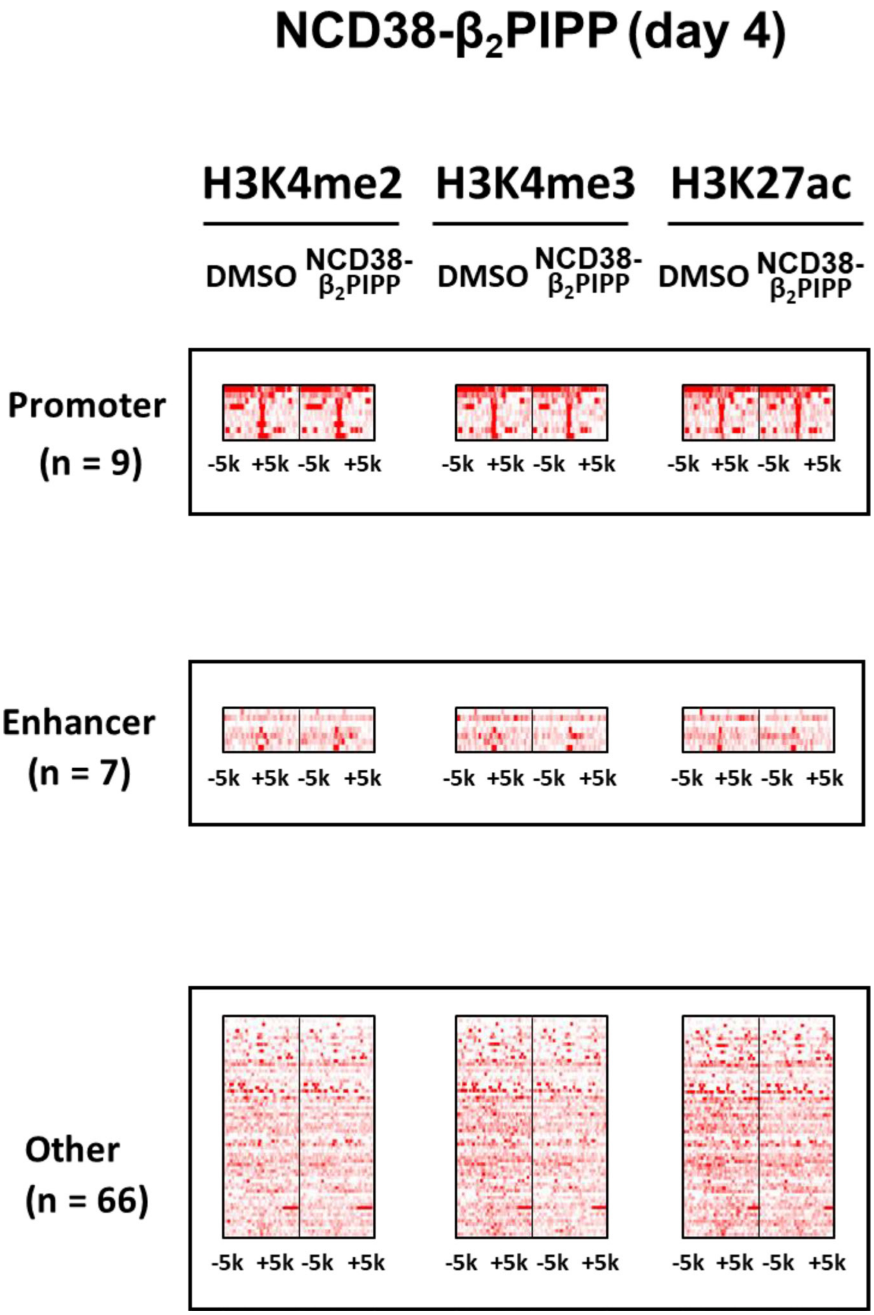

Supplementary Figure 16: Alteration of H3K27ac by NCD38-β_2PIPP treatment for four days. Heatmap showed H3K27ac-increased regions in promoter, enhancer, and other regions observed on day 30 (Figure 10B). The alteration was not so observed on day 4.

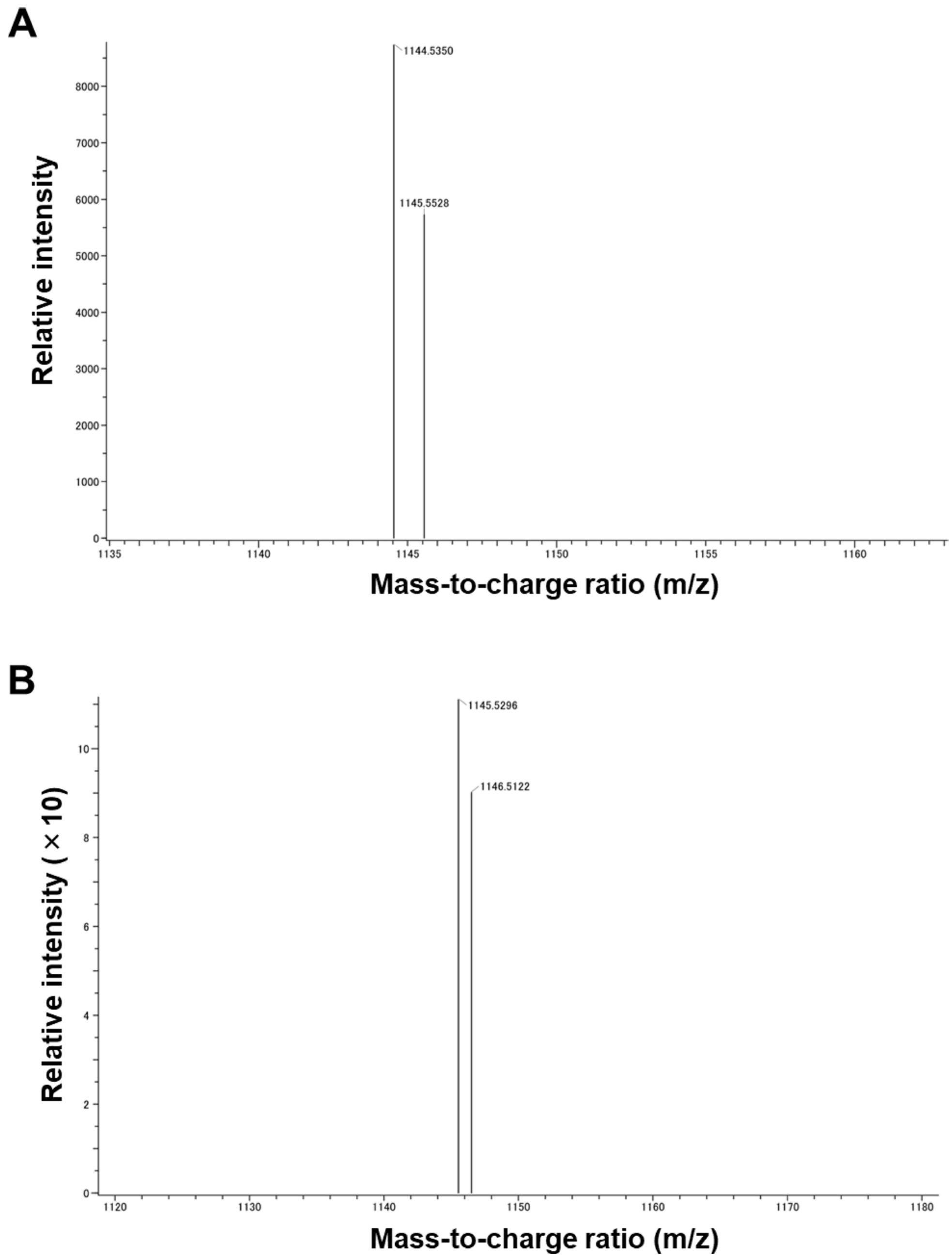

Supplementary Figure 17: Positive-ion mass spectra for the conjugates. (**A**) NCD38-β_2P_4. The molecular weight was calculated to be 1144.5363, and found to be 1144.5350. (**B**) NCD38-β_2PIPP. The molecular weight was calculated to be 1145.5316, and found to be 1145.5296.

Supplementary Table 1: Sequences of oligo DNA for EMSA

Oligo sequence	Length of dsDNA (bp)
**WWWWWW**	
WWWWWW_F: 5′-FAM-GGAGGATATAAGGAGG-3′	16
WWWWWW_R: 5′-CCTCCTTATATCCTCC-3′	
**WWCGWW**	
WWCGWW_F: 5′-FAM-GGAGGATCGAAGGAGG-3′	16
WWCGWW_R: 5′-CCTCCTTCGATCCTCC-3′	
**GWWWWG**	
GWWWWG_F: 5′-FAM-GGAGGGTATAGGGAGG-3′	16
GWWWWG_R: 5′-CCTCCCTATACCCTCC-3′	
**WWWWWWWW**	
WWWWWWWW_F: 5′-FAM-GGAGGATTATATAGGAGG-3′	18
WWWWWWWW_R: 5′-CCTCCTATATAATCCTCC-3′	
**GWCGWG**	
GWCGWG_F: 5′-FAM-GGAGGGTCGAGGGAGG-3′	16
GWCGWG_R: 5′-CCTCCCTCGACTCC-3′	
**GWGCWG**	
GWGCWG_F: 5′-FAM-GGAGGGTGCAGGGAGG-3′	16
GWGCWG_R: 5′-CCTCCCTGCACCCTCC-3′	
**WWWGCWWW**	
WWWGCWWW_F: 5′-FAM-GGAGGTATGCAATGGAGG-3′	18
WWWGCWWW_R: 5′-CCTCCATTGCATACCTCC-3′	
